# Mild Angle Early Onset Idiopathic Scoliosis Children Avoid Progression Under FITS Method (Functional Individual Therapy of Scoliosis)

**DOI:** 10.1097/MD.0000000000000863

**Published:** 2015-05-22

**Authors:** Marianna Białek

**Affiliations:** FITS Center, Jawor, Poland (MB).

## INTRODUCTION

Infantile Idiopathic Scoliosis and Juvenile Idiopathic Scoliosis with the onset before the age of 10 years are named Early Onset Idiopathic Scoliosis (EOIS). This pathology is very difficult to treat due to the very long period of growth of the child to the moment of biological maturity and the related risk of progression of the curvature. The aim of treatment in young children with idiopathic scoliosis is to maintain for years the curve at low-angle value in order to make the spine growth possible in the best conditions of minimal asymmetry. Thus, the development of the vicious circle of scoliosis progression^1^ is hoped to be prevented.

Physiotherapy for the stabilization of idiopathic scoliosis angle in growing children remains controversial. So far, little data on the effectiveness of physiotherapy in children with EOIS were published.^2,3^

The Functional Individual Therapy for Scoliosis (FITS method) was introduced by Białek and M’hango, described in 2004, then published in 2008^4^ and in 2010.^5^ The FITS method comprises 3 stages: examination (stage I), facilitation of correction (stage II), and 3-dimensional (3D) correction (stage III). The results in adolescent patients were previously reported.^6^ This study aims to assess the early results in children who started therapy before the age of 10 years for EOIS.

## MATERIAL AND METHOD

### Patients

**FIGURE 1:**
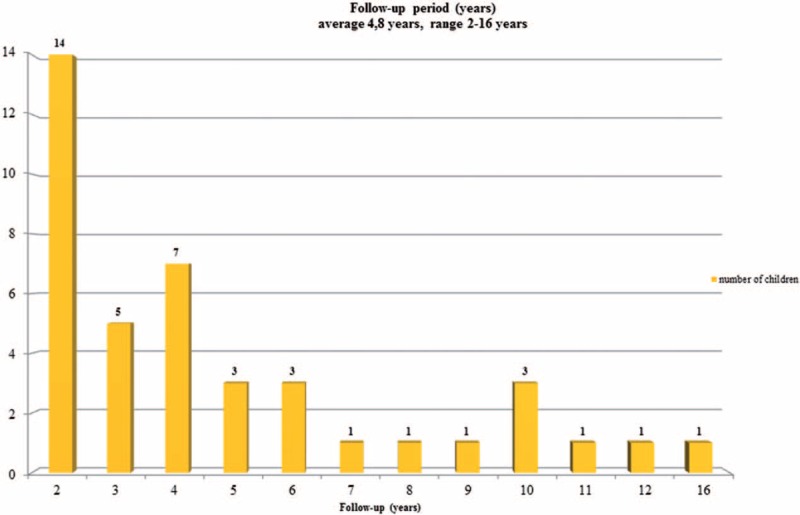
Follow-up period.

The charts of the patients archived in a prospectively collected database were retrospectively reviewed. The inclusion criteria to this study were as follows: diagnosis of EOIS based on clinical examination and spine radiography, age below 10 years at the beginning of treatment, both girls and boys, Cobb angle between 11° and 30°, Risser zero, FITS therapy, no other treatment in the past or during FITS (like nighttime bracing), and follow-up period minimum 2 years from the initiation of the FITS treatment (Figure 1).

**FIGURE 2:**
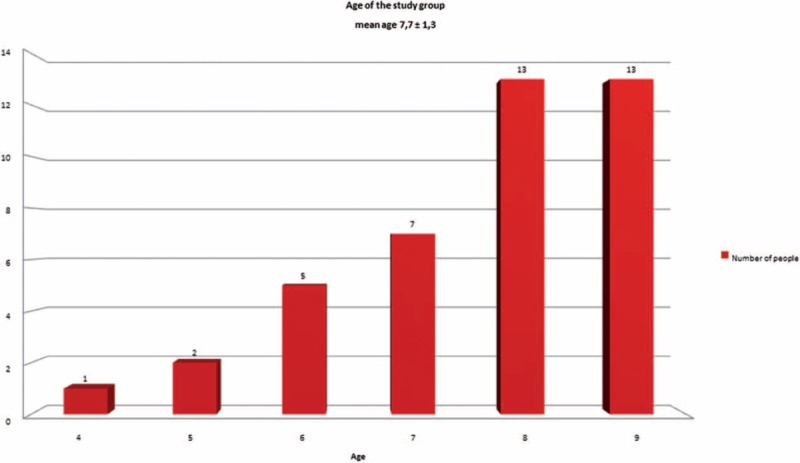
Age of the study group.

There were 41 children who met these inclusion criteria and underwent FITS treatment for EOIS: 36 girls and 5 boys, mean age of 7.7 ± 1.3 years (range from 4 to 9 years) (Figure 2).

The patients presented curve pattern: A1—single thoracic (5 children), A2—single thoracolumbar (22 children), and A3—double thoracic/thoracolumbar (14 children), totally 55 structural curvatures (Table 1).

**TABLE 1:**
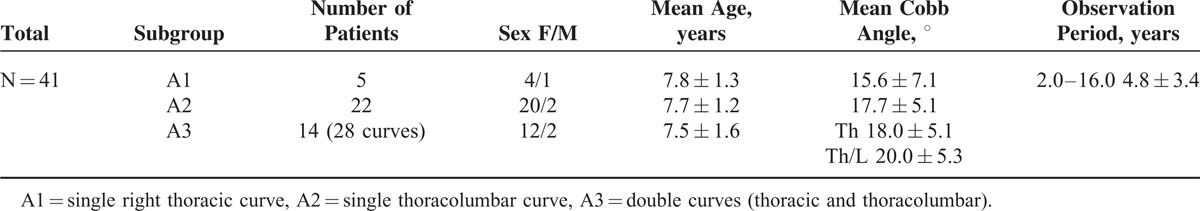
Initial Characteristics of the Patients

### Examination

All children were examined by the author. The history comprising age of diagnosis, family history and developmental history were investigated. The clinical assessment comprised classical orthopedic examination completed with detailed examination performed according to FITS method principles.^7^ The X-ray in standing position (not older than 6 months) was measured.

**FIGURE 3:**
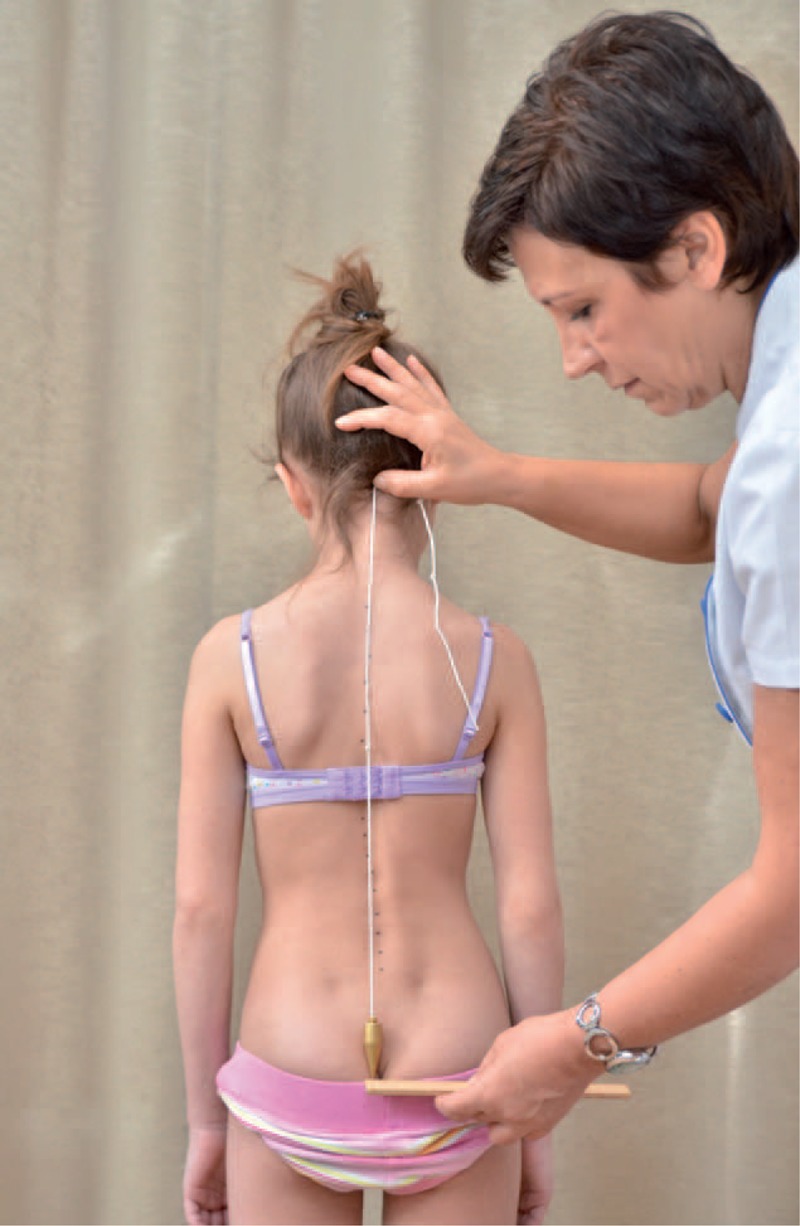
Distance from plumb line to anal cleft.

**FIGURE 4:**
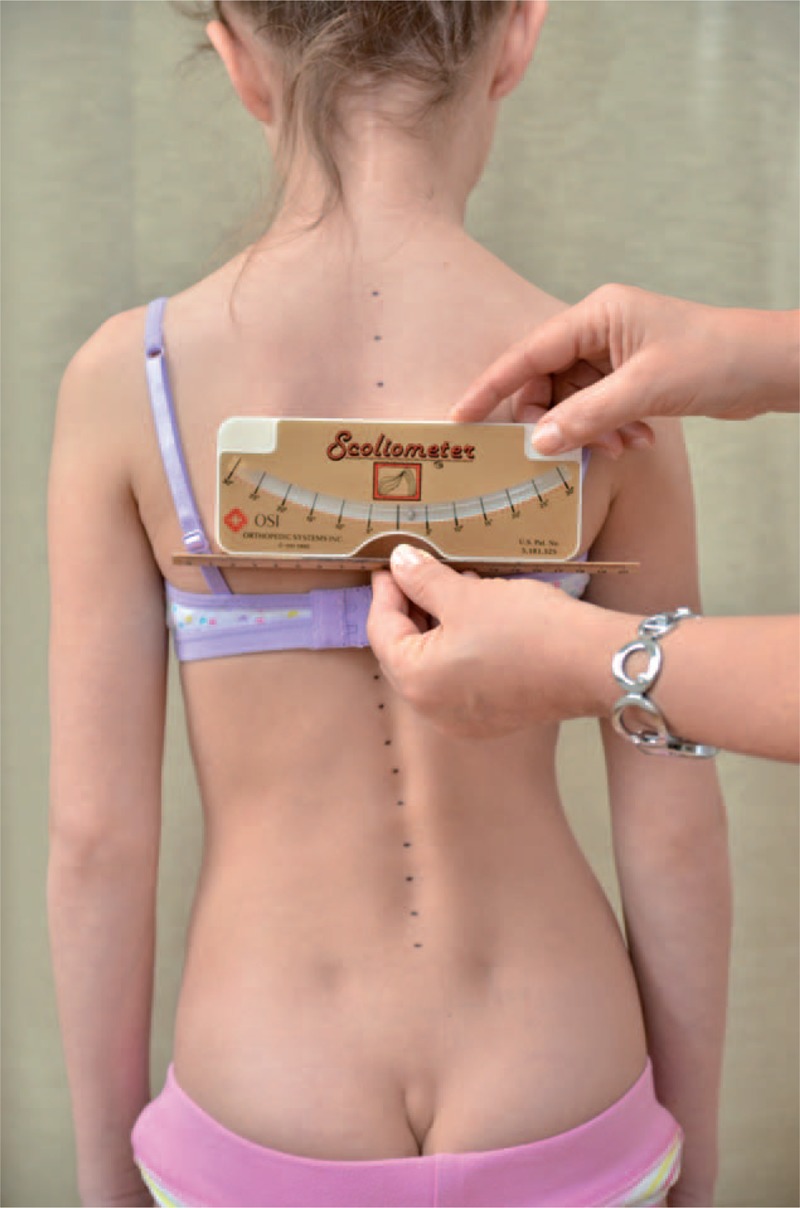
Checking scapulas level.

**FIGURE 5:**
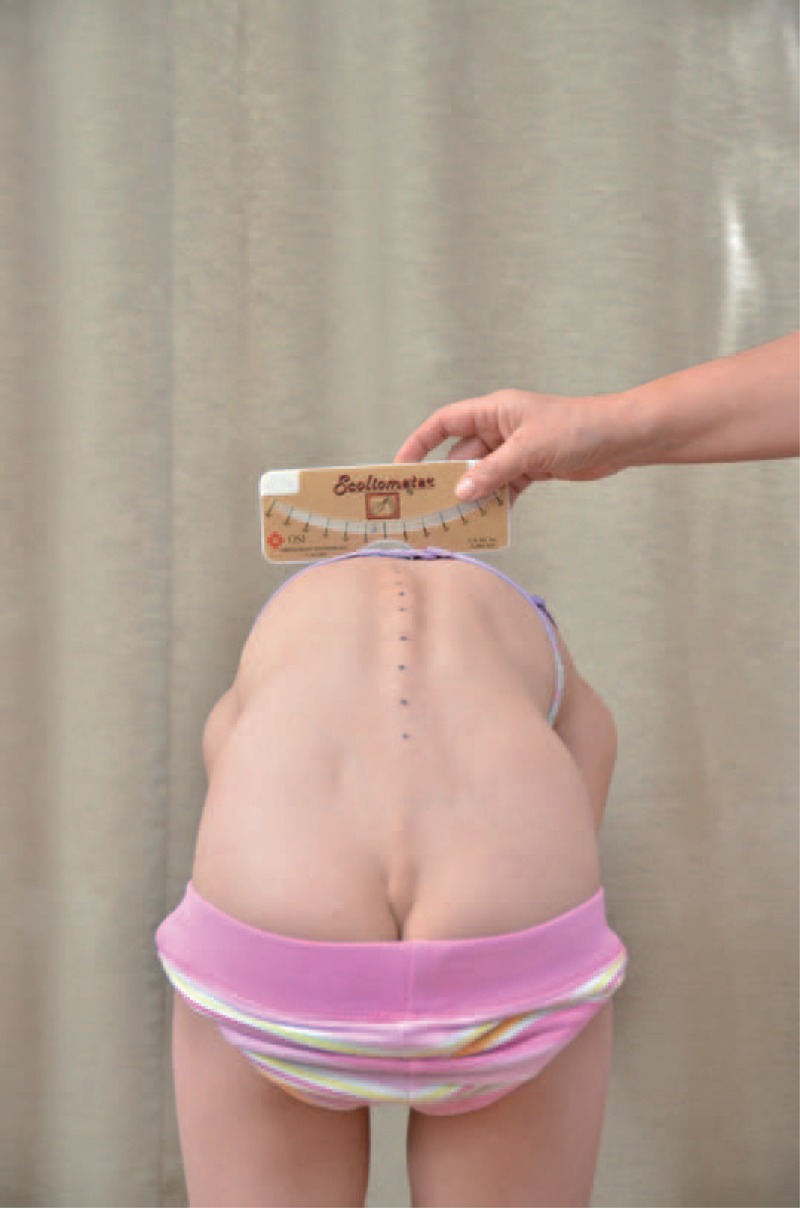
Measurement of the angle of trunk rotation at thoracic level.

**FIGURE 6:**
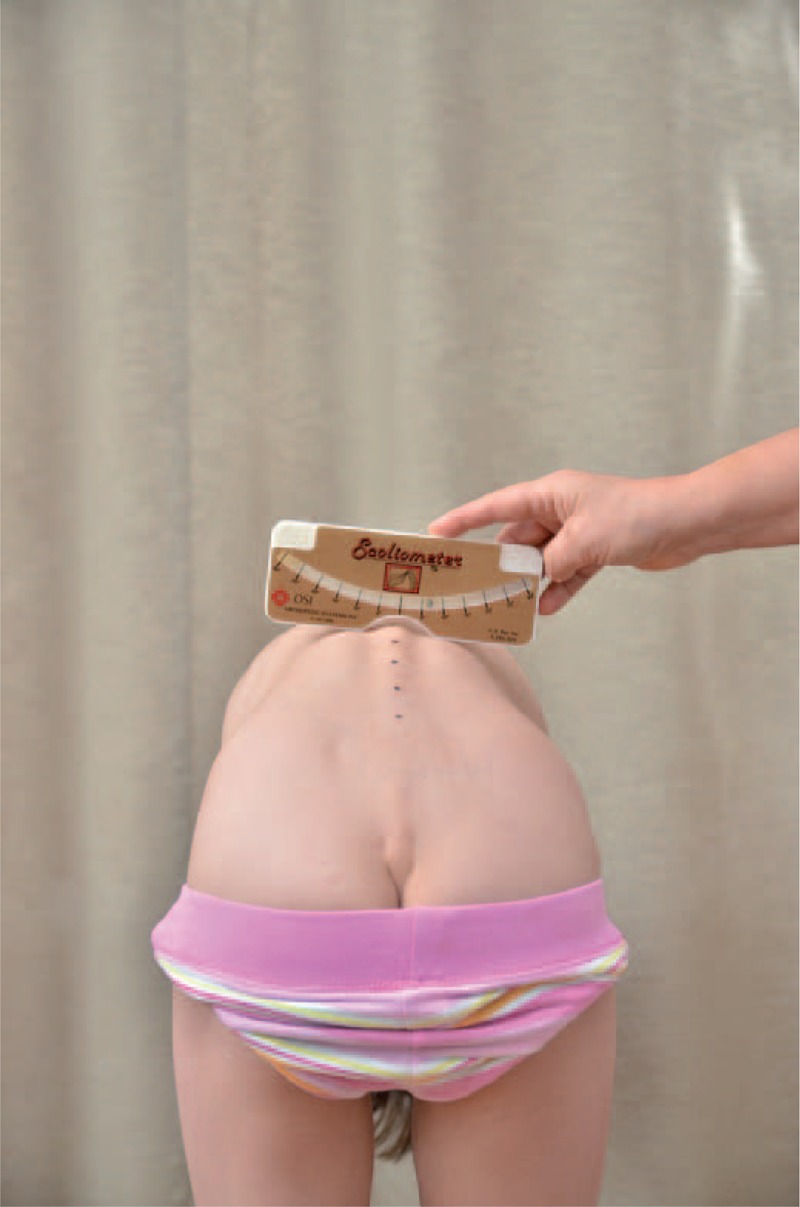
Measurement of the angle of trunk rotation at lumbar level.

The classical examination included:

1. distance from plumb line to: anal cleft, apex of primary curve, apex of secondary curve, and left and right scapula edge (Figure 3);
2. checking scapulas level with scoliometer (Figure 4); and
3. measurement of the angle of trunk rotation at thoracic (Figure 5) and at lumbar level (Figure 6) using Bunnell scoliometer.

**FIGURE 7:**
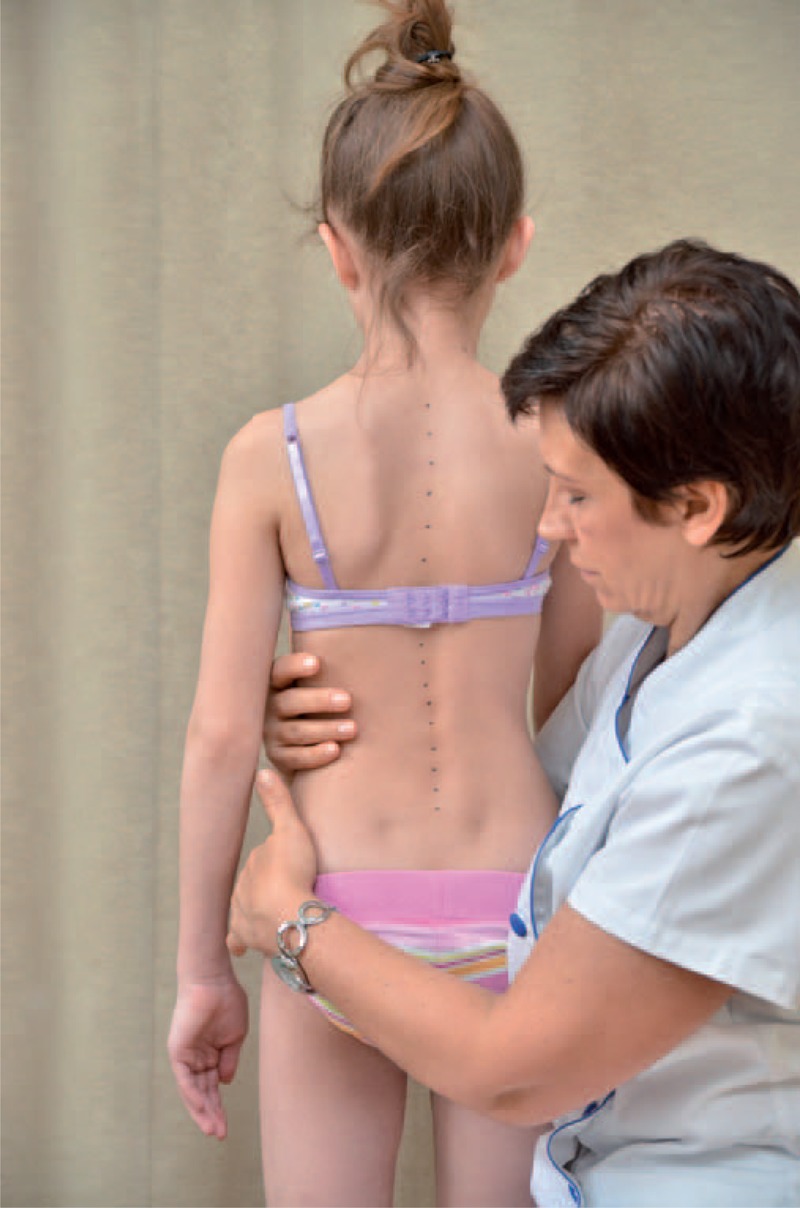
Assessment of possibilities for scoliosis correction in standing position—before the test.

**FIGURE 8:**
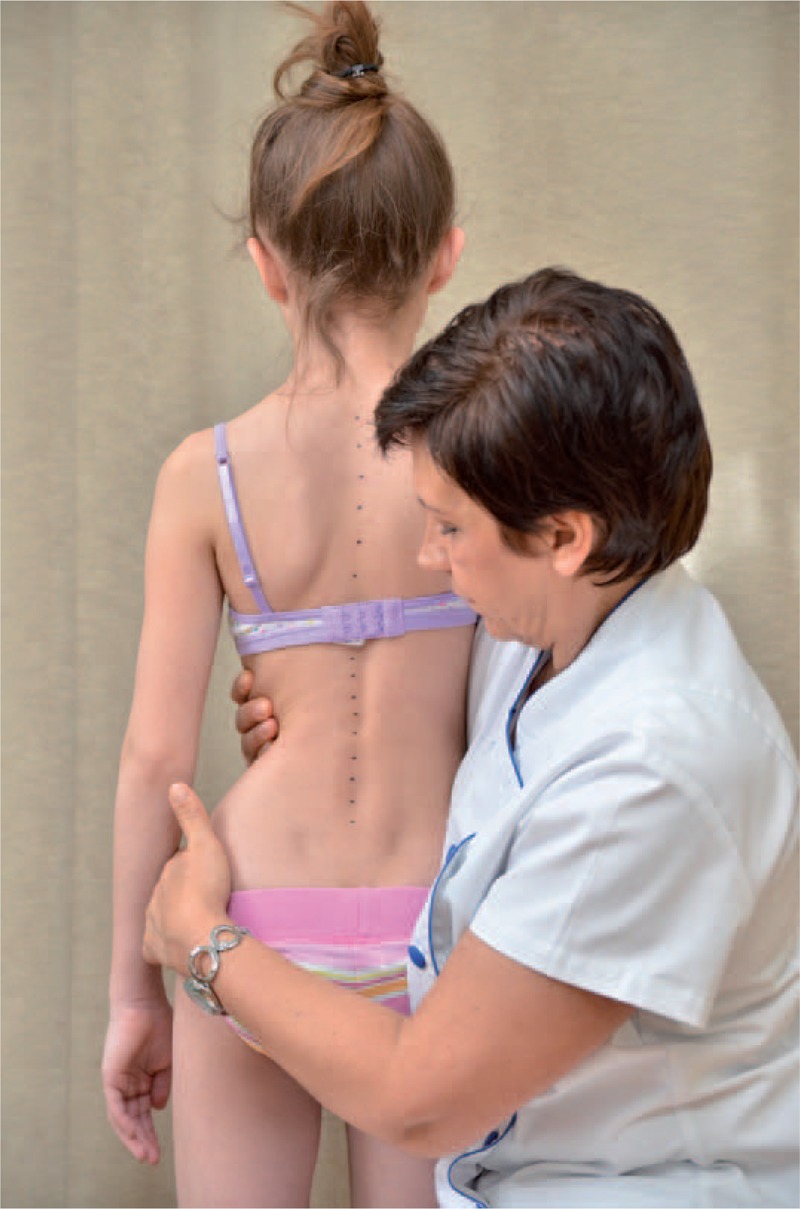
Assessment of possibilities for scoliosis correction in standing position—during the test.

**FIGURE 9:**
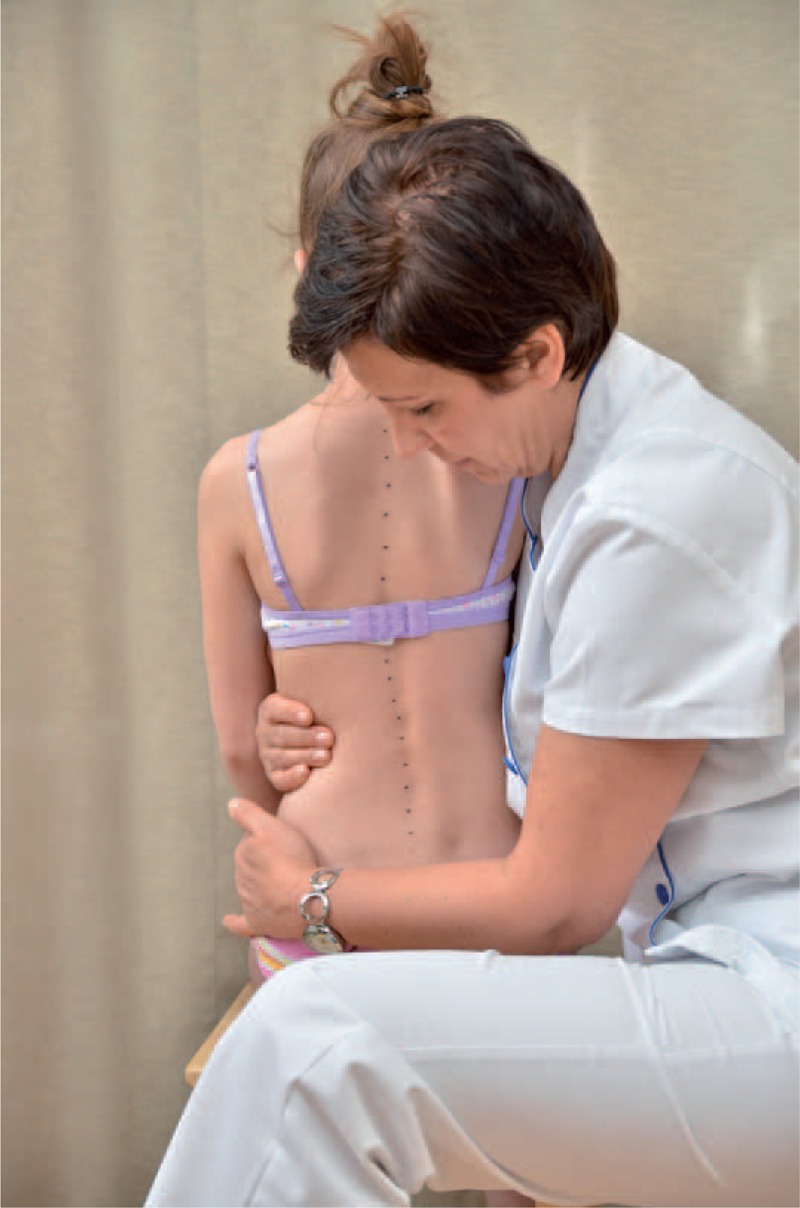
Assessment of possibilities for scoliosis correction in sitting position—before the test.

**FIGURE 10:**
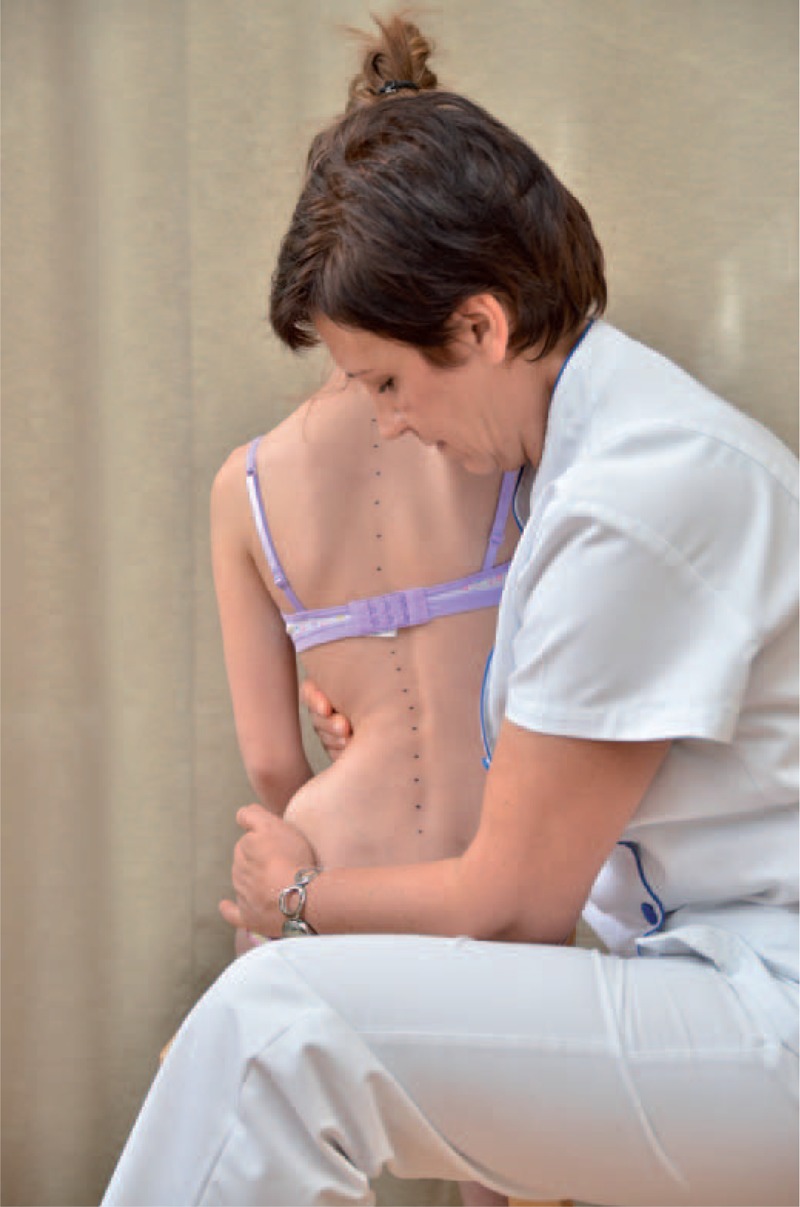
Assessment of possibilities for scoliosis correction in sitting position—during the test.

**FIGURE 11:**
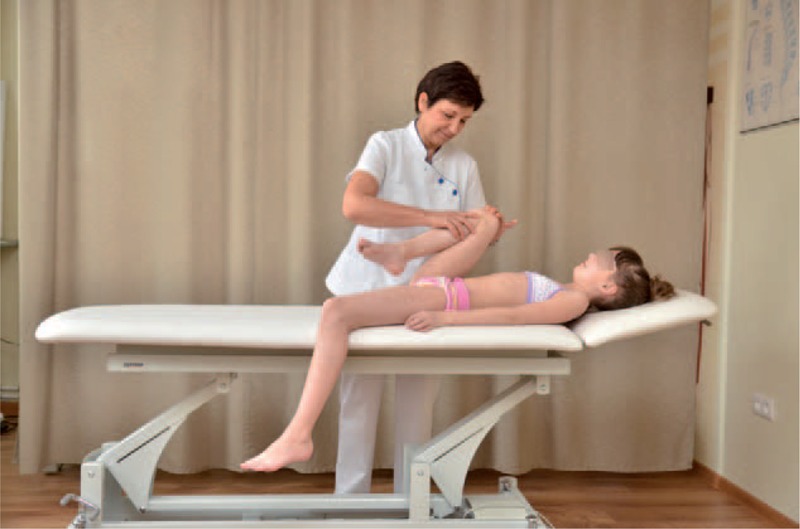
Adductor magnus testing.

**FIGURE 12:**
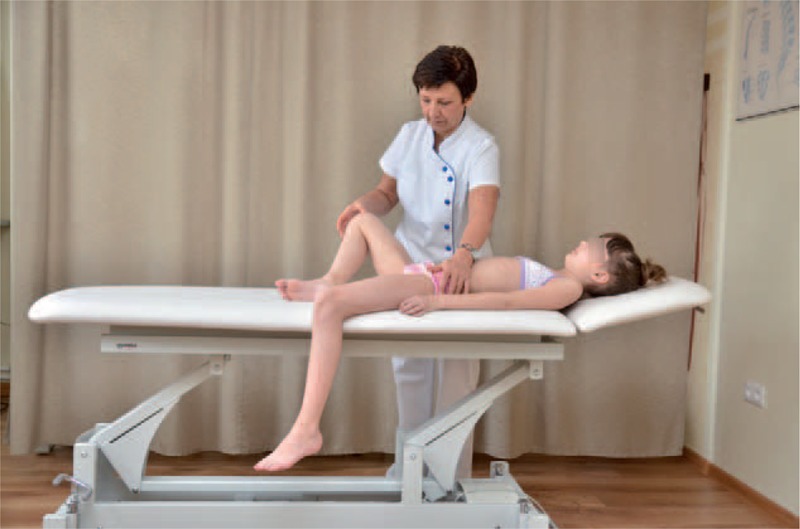
Adductor longus testing.

**FIGURE 13:**
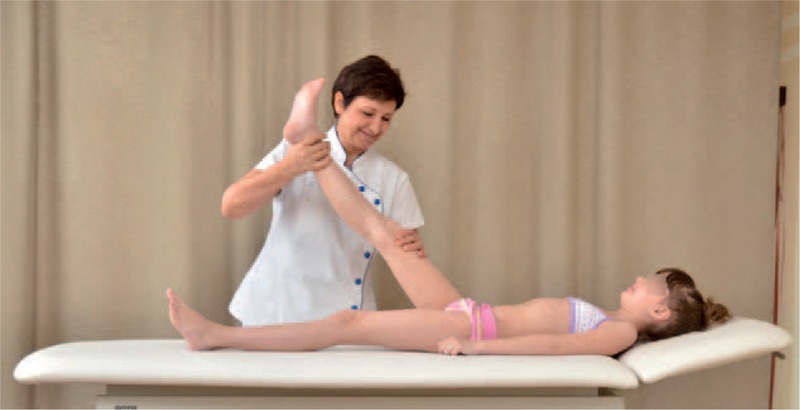
Hamstrings testing.

The FITS examination included additionally:

1. exam for the leg length inequality;
2. assessment of the lower limbs in standing and gait;
3. observation of type and location of compensation;
4. assessment of possibilities for scoliosis correction in standing (Figures 7 and 8) and sitting position (Figures 9 and 10); and
5. assessment of the length of muscles in lower limbs and pelvis particularly involved in postural asymmetries in young children, namely adductor longus, adductor magnus, and hamstrings (Figures 11–13).

Although testing possibility of scoliosis correction by making corrective movement, the therapist is able to feel which myofascial structures should be addressed first. In order to indicate the direction of therapy and assess effectiveness of therapeutic procedures, we test corrective movement during each session. Corrective movement at the beginning of therapy can be done only in 1 plane: shift, rotation, or flexion/extension. In further stages of therapy 3D corrective movement should be included.

The clinical parameters at study baseline are presented in Table 2.

**TABLE 2:**
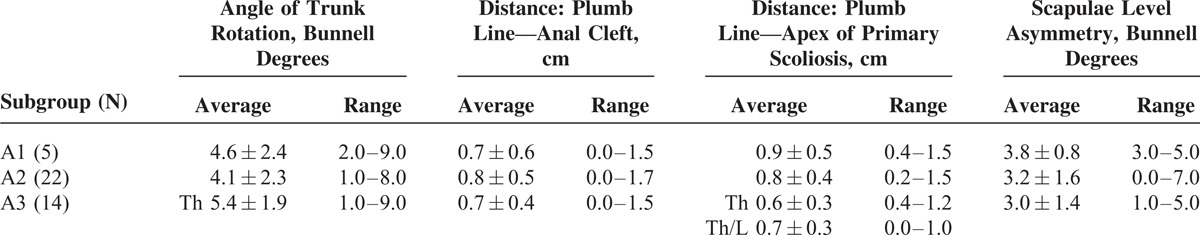
Clinical Parameters at Study Entry

### Course of FITS Therapy

We start with relaxation of structures restricting scoliosis correction by using physiotherapeutic techniques described elsewhere like: contract-relax technique, passive and active myofascial release,^8,9^ trigger points, and^10^ joint mobilization.^11,12^

These techniques are addressed in the area of myofascial bands described by Myers:^13^

- SBL (superficial back line),
- DFL (deep front line),
- LL (lateral muscle line),
- SL (spiral muscle line), and
- SFL (superficial front line).

In case the examination revealed no soft tissue restriction and especially when the child presented with soft tissue laxity and joint hypermobility^14^—the stage II of the FITS method could be omitted and the therapist went directly to stage III (3D correction).

**FIGURE 14:**
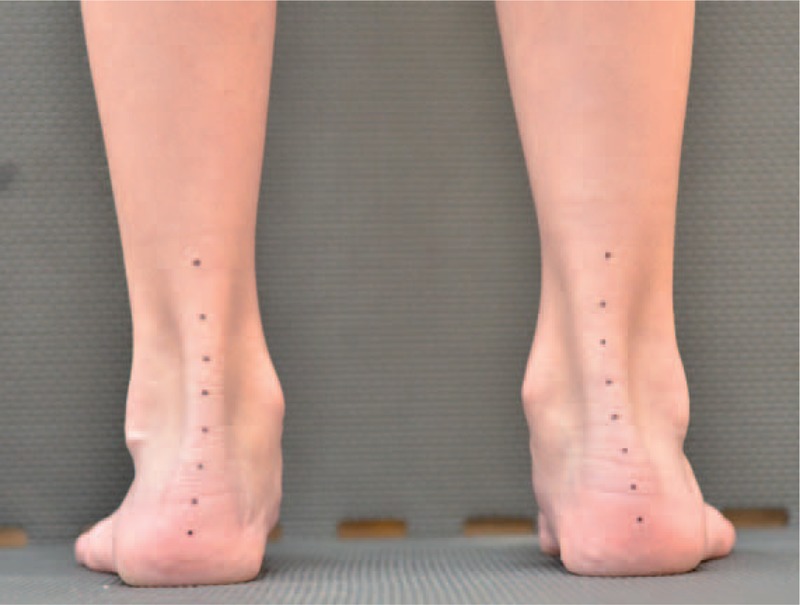
Spontaneous loading of the feet.

**FIGURE 15:**
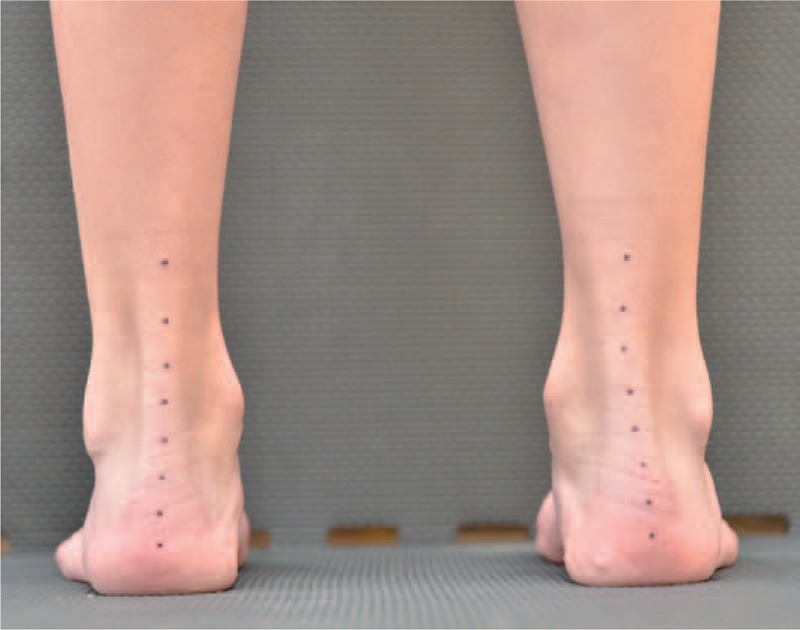
Loading in correction.

To build and stabilize new corrective patterns of posture in functional positions we started from correct foot loading using sensory motor balance training according to Greenman^15^ (Figures 14 and 15).

**FIGURE 16:**
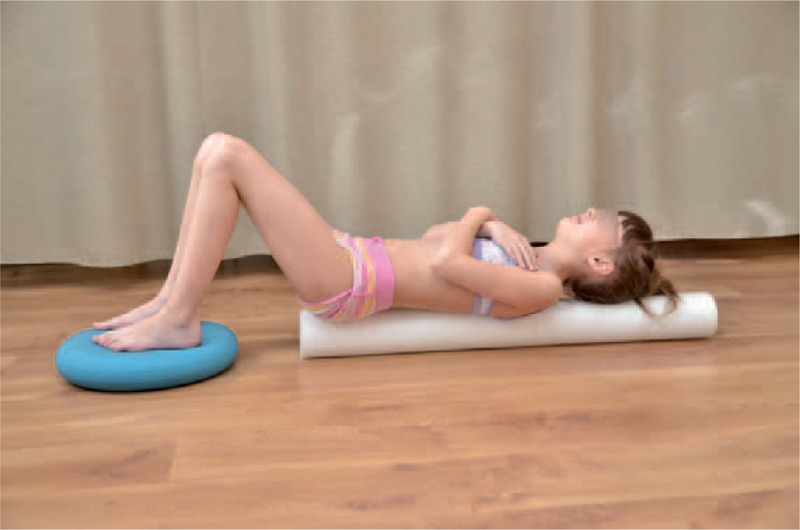
Exercise of lower trunk stabilization on a roll and sensorimotor pillows.

**FIGURE 17:**
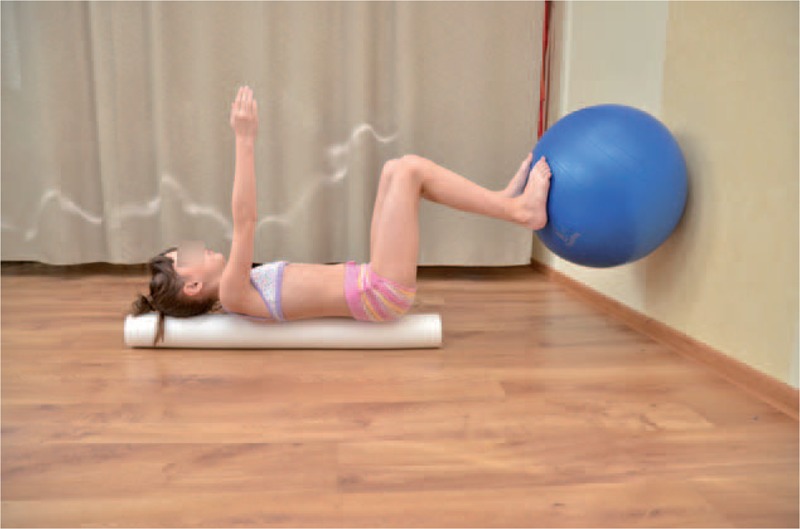
Exercise of lower trunk stabilization on a roll and the ball.

Observing young children with scoliosis we noticed unsettled stabilization of the lower part of trunk, especially during everyday activities. Stabilization exercise for this lower part of the trunk was essential for the performance of corrective patterns of the upper part of trunk and shoulder girdle (Figures 16 and 17).

Facilitation to 3-plane corrective breathing was done after diaphragm release and restoring the joint mobility in thoracic spine for the thorax derotation breathing exercise. The effectiveness of the mentioned exercise can be improved by adding elongation of scoliosis concavity by using upper and lower limb patterns. The exercise was an essential element of costal hump correction.

The teaching of corrective patterns was done in open and closed kinematic chain exercise, with the use of Thera-Band. Each limb pattern consisted of correction in sagittal, frontal, and transverse plane. The choice of each element of every corrective pattern depended on Cobb angle, size and direction of trunk rotation, position of the spine in sagittal plane, and location of functional compensation.^6,7^

These patterns were held until the moment when on the concave side of the primary curve (above and below this curve) the minor functional compensation appeared—of less than 3–4 degree of rotation. This compensation concerned only soft tissues, not structures seen on X-rays. At this moment changing of patterns for upper extremities should be done in the direction to elongate both sides.

During the course of FITS treatment the children received individual treatment twice a month (45–60 minutes) at the beginning period. This therapy was performed by the author herself. Between the individual therapy meetings, the patients performed adequately selected and prescribed set of exercises at home, once a day (30–45 minutes). In cases the FITS educated physiotherapists were accessible at proximity, the patients received individual treatment at their places of residence. The patients were also educated to sit in a correct position during classes at school and at homework. Twice a year, a 2-week in-patient physiotherapy was offered in the form of a winter or summer rehabilitation camp. Five children out of 41 analyzed in this study did participated in at least 1 2-week rehabilitation camp.

**FIGURE 18:**
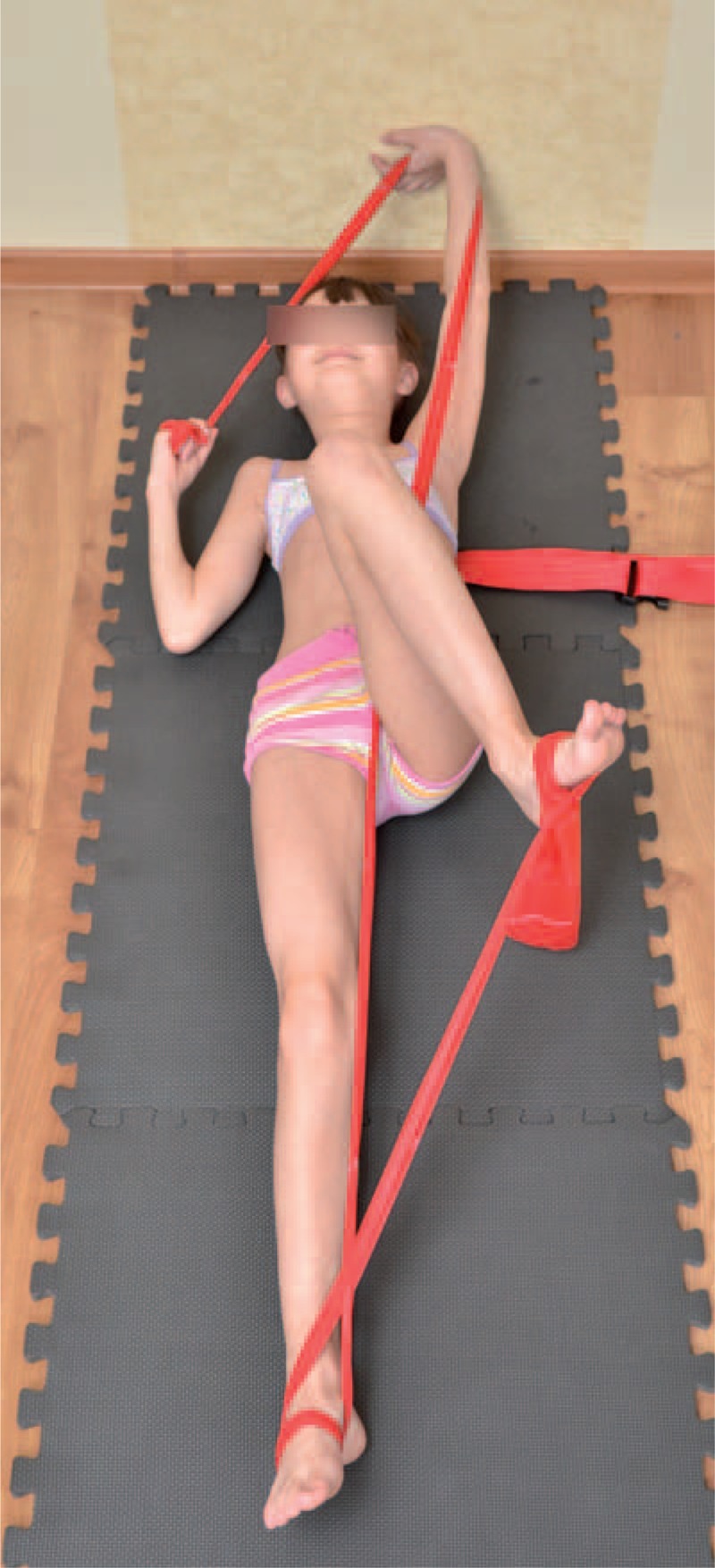
An example of corrective pattern in supine position.

**FIGURE 19:**
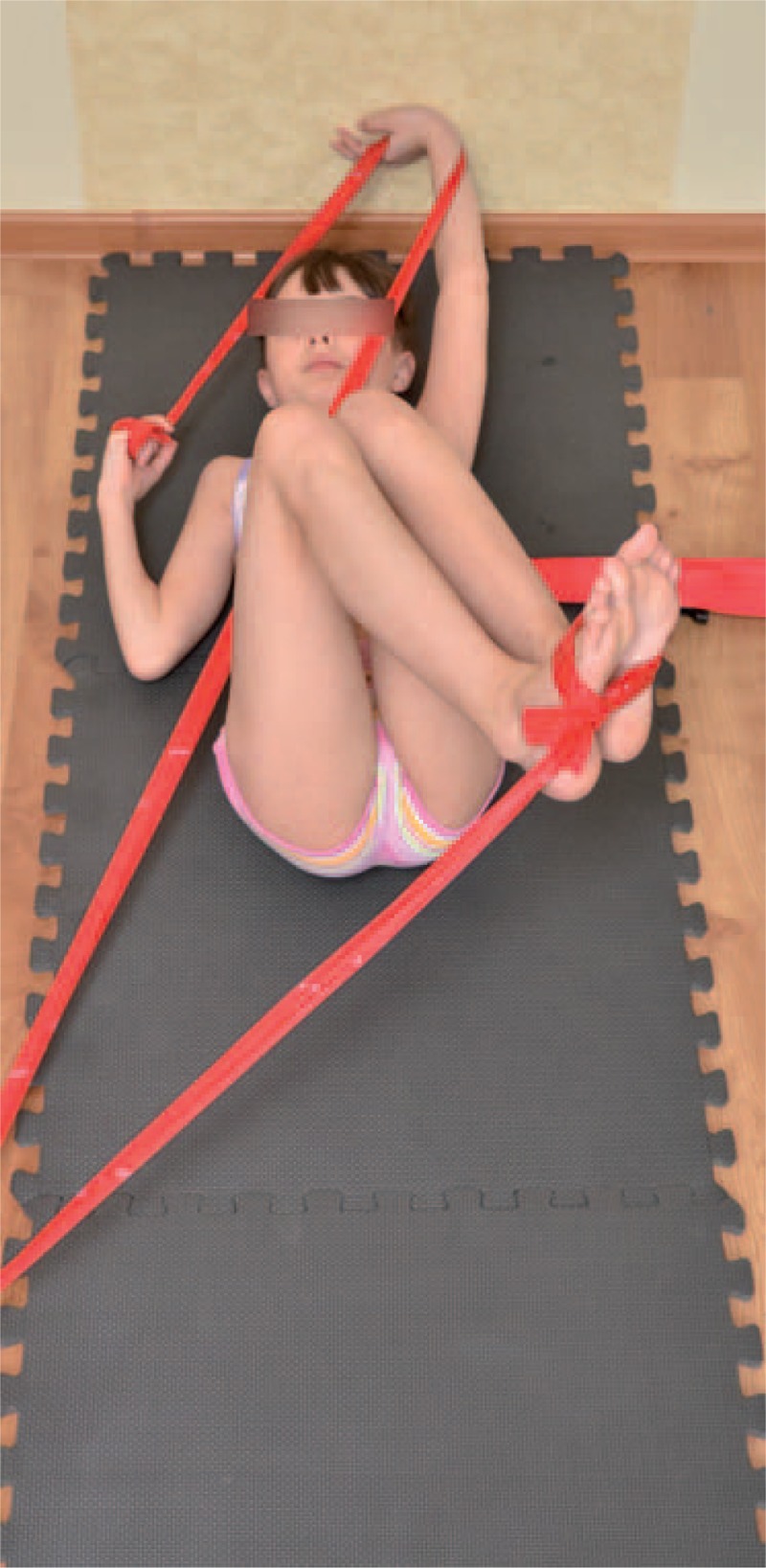
Alternative way of exercises to Figure 18.

**FIGURE 20:**
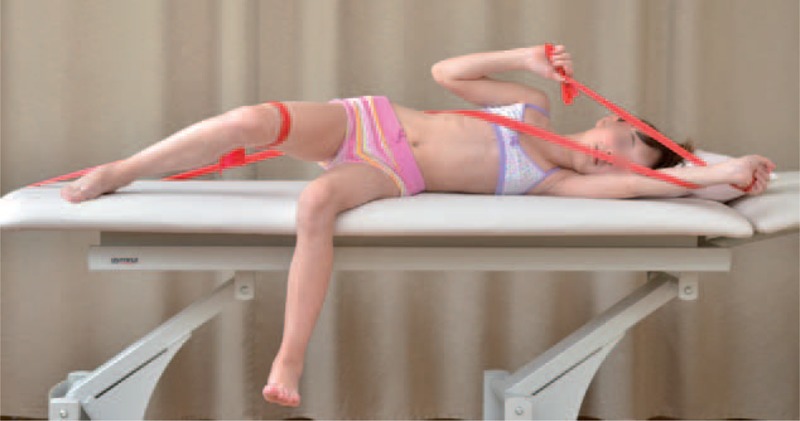
An example of corrective pattern on the left side—front view.

**FIGURE 21:**
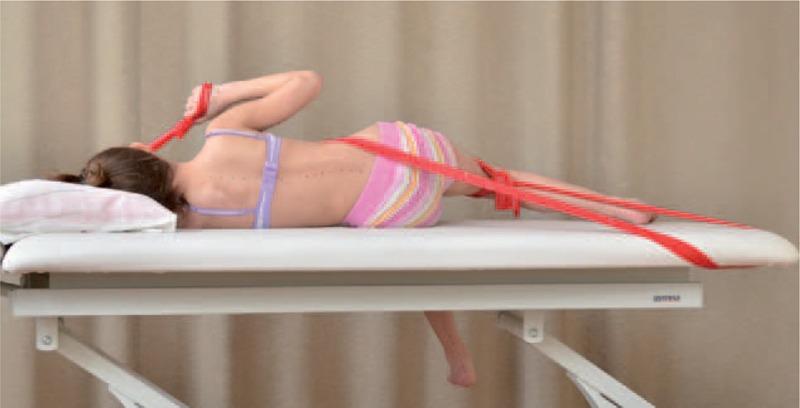
An example of corrective pattern on the left side—back view.

**FIGURE 22:**
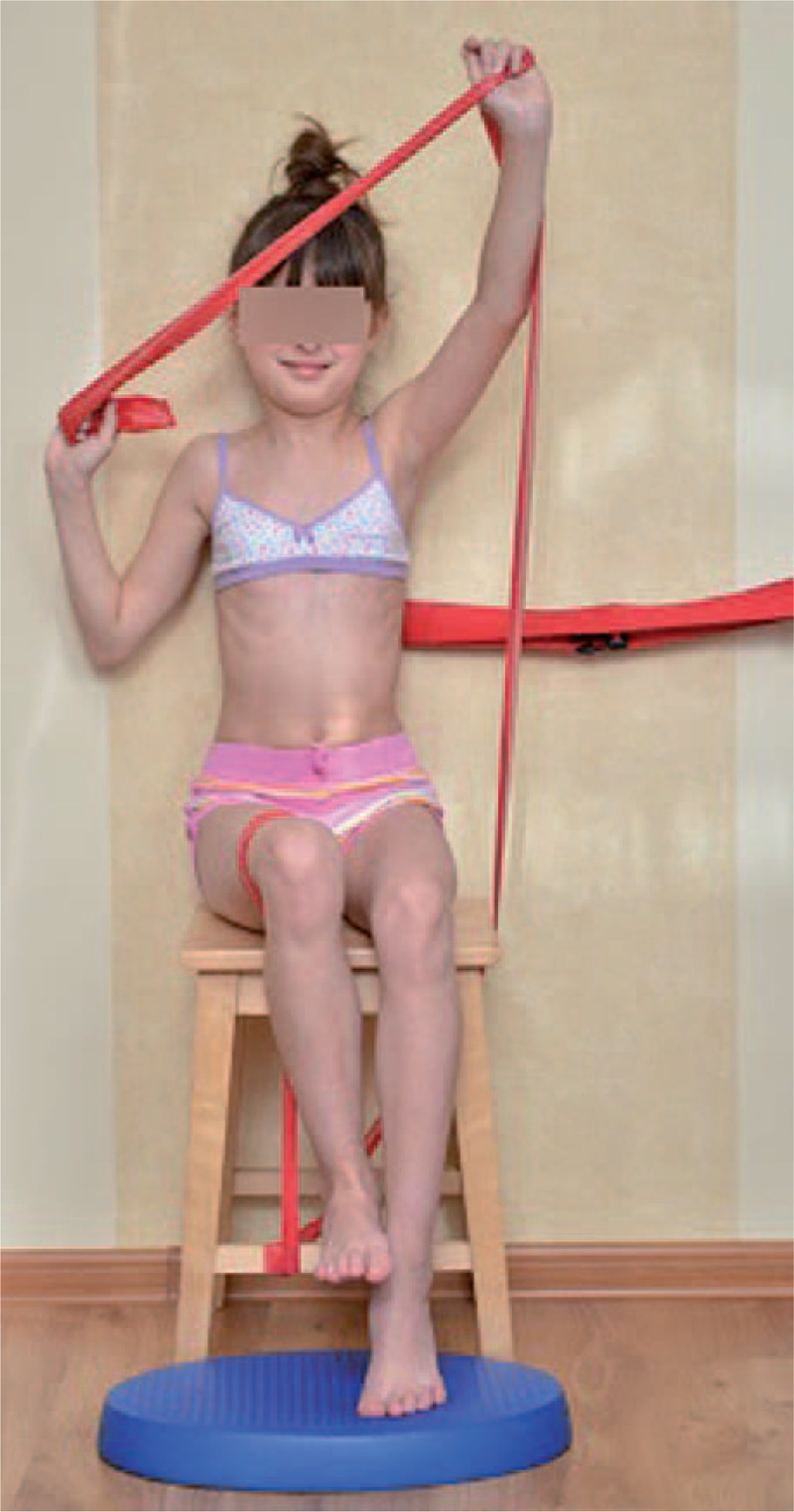
An example of corrective pattern in sitting position.

Sample exercises for scoliosis Thdex and Th/L sin shows figures below (Figures 18–22).

### Re-evaluation of Patients

At follow-up, all clinical parameters were reassessed and the patients were subjected to X-ray analysis for the Cobb angle. The patients had the X-rays taken in the place where they lived. Cobb angle was measured by the treating physician. The percentage of children in whom the Cobb angle decreased by more than 5°, the percentage of children in whom the Cobb angle was stable during the observation period (angle ± 5°), and the percentage of children in whom the Cobb angle increased by more than 5° were calculated at follow-up.

## RESULTS

The minimum follow-up was 2 years after initiation of the FITS treatment, the maximum was 16 years, mean 4.8 years. At follow-up the mean age was 12.5 ± 3.4 years (range 8–20 years). Out of 41 children, 10 passed pubertal growth spurt at the final follow-up while 31 were still immature and continued FITS therapy. Out of 41 children, 27 improved, 13 were stable and 1 progressed. Out of 55 structural curves, 32 improved, 22 were stable and 1 progressed. Seven of 41 children were lost from observation after 2 years of FITS therapy, and no further data concerning their Cobb angle are available. At the moment these 7 children were lost from the follow-up, their mean age was 10 ± 1, range 9 to 12 years. The values of the measured parameters before FITS therapy and at follow-up are presented in Table 3.

**TABLE 3:**
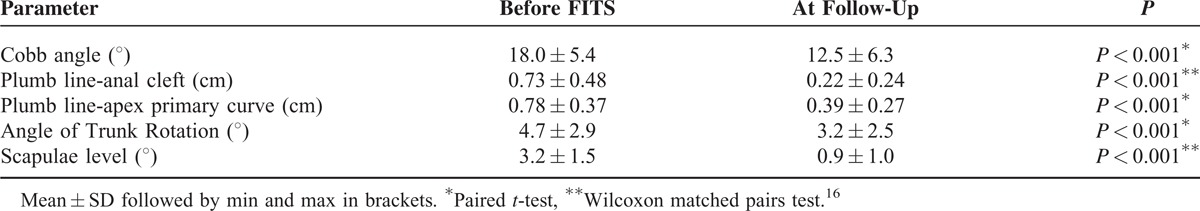
Comparison of Pre- and Post-Treatment Values of Measured Parameters for the Total N = 41 Patients, 55 Curvatures

The percentages of curve correction, stabilization, and progression at follow-up are presented in Table 4.

**TABLE 4:**
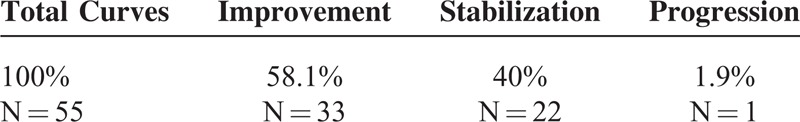
Percentage Values of Scoliosis Improvement, Stabilization and Progression

Example X-ray patient—Aleksandra, who started FITS therapy in 2008, at the age of 8 years and follow-up (Table 5, Figures 23–30).

**TABLE 5:**
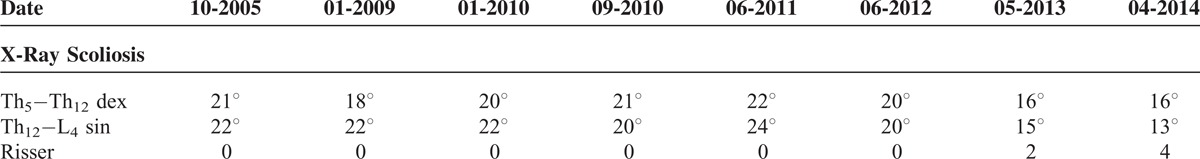
X-Ray in Subsequent Years of Functional Individual Therapy of Scoliosis

**FIGURE 23:**
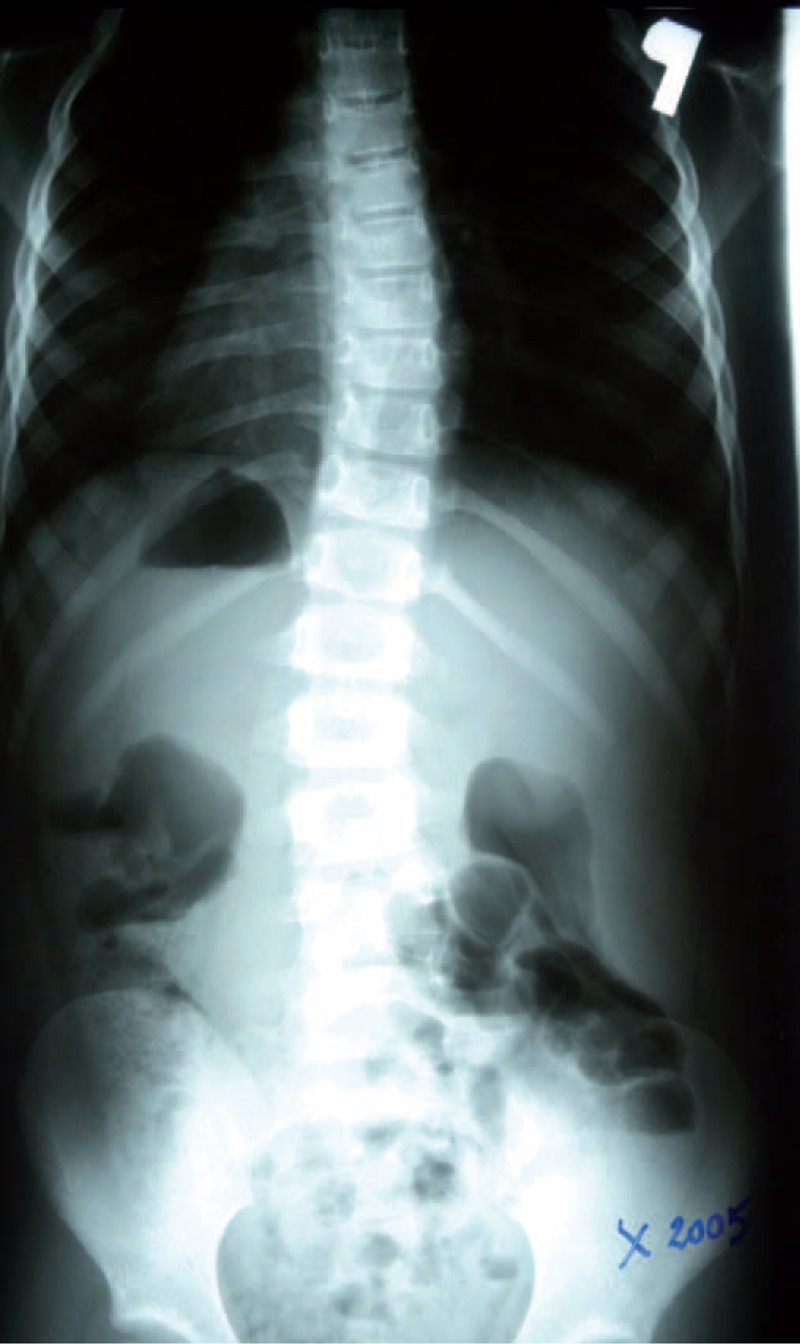
X-ray 10-2005.

**FIGURE 24:**
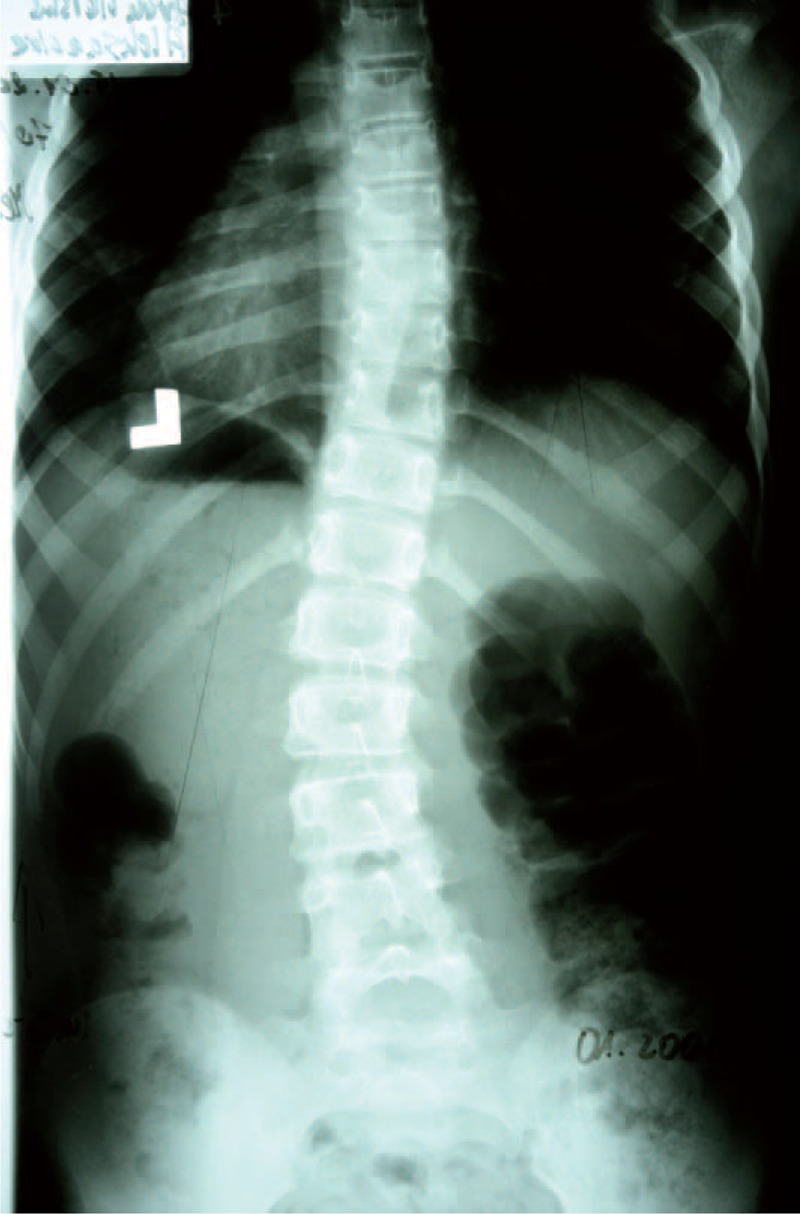
X-ray 01-2008.

**FIGURE 25:**
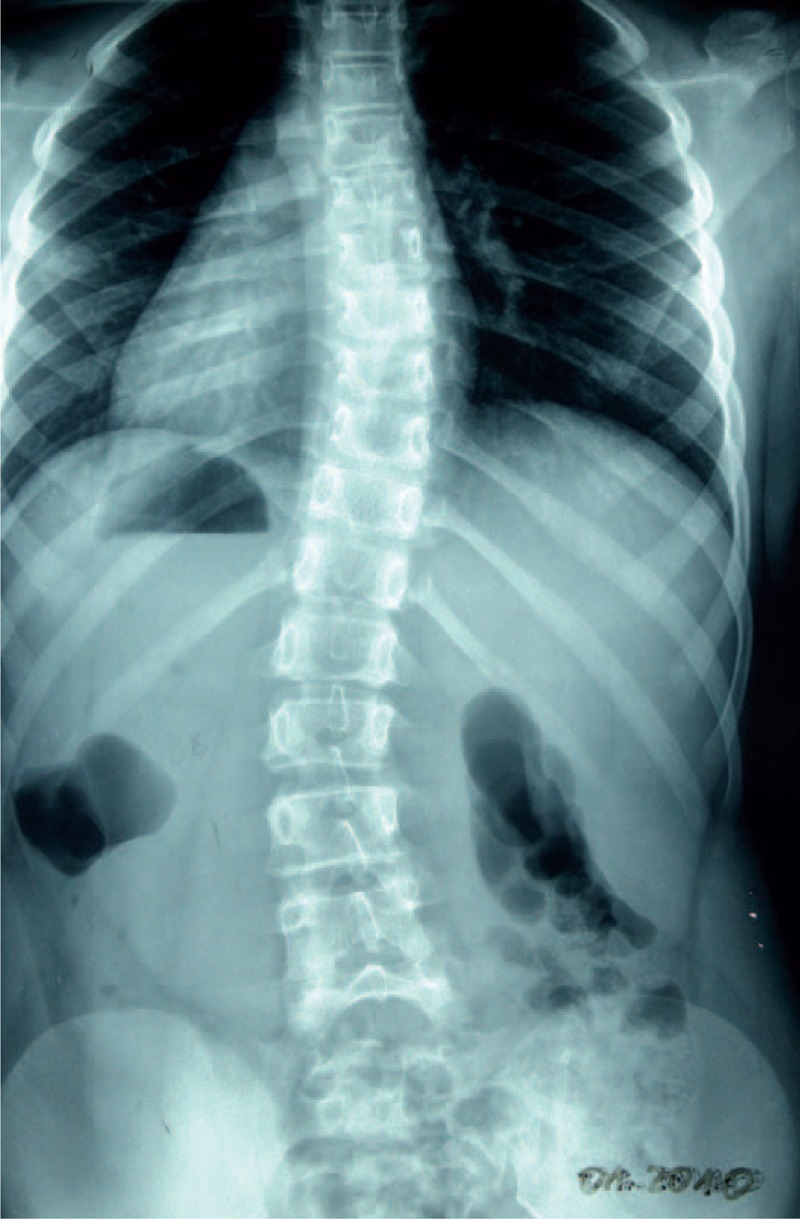
X-ray 01-2010.

**FIGURE 26:**
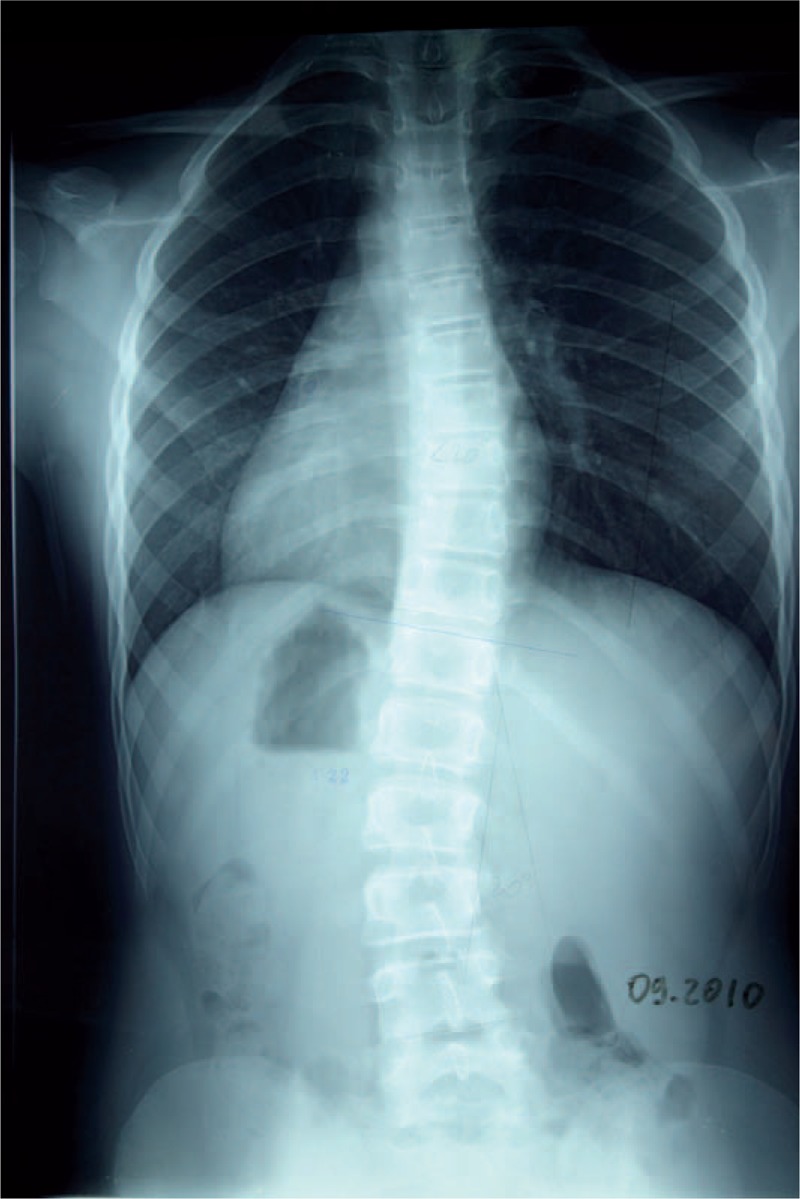
X-ray 09-2010.

**FIGURE 27:**
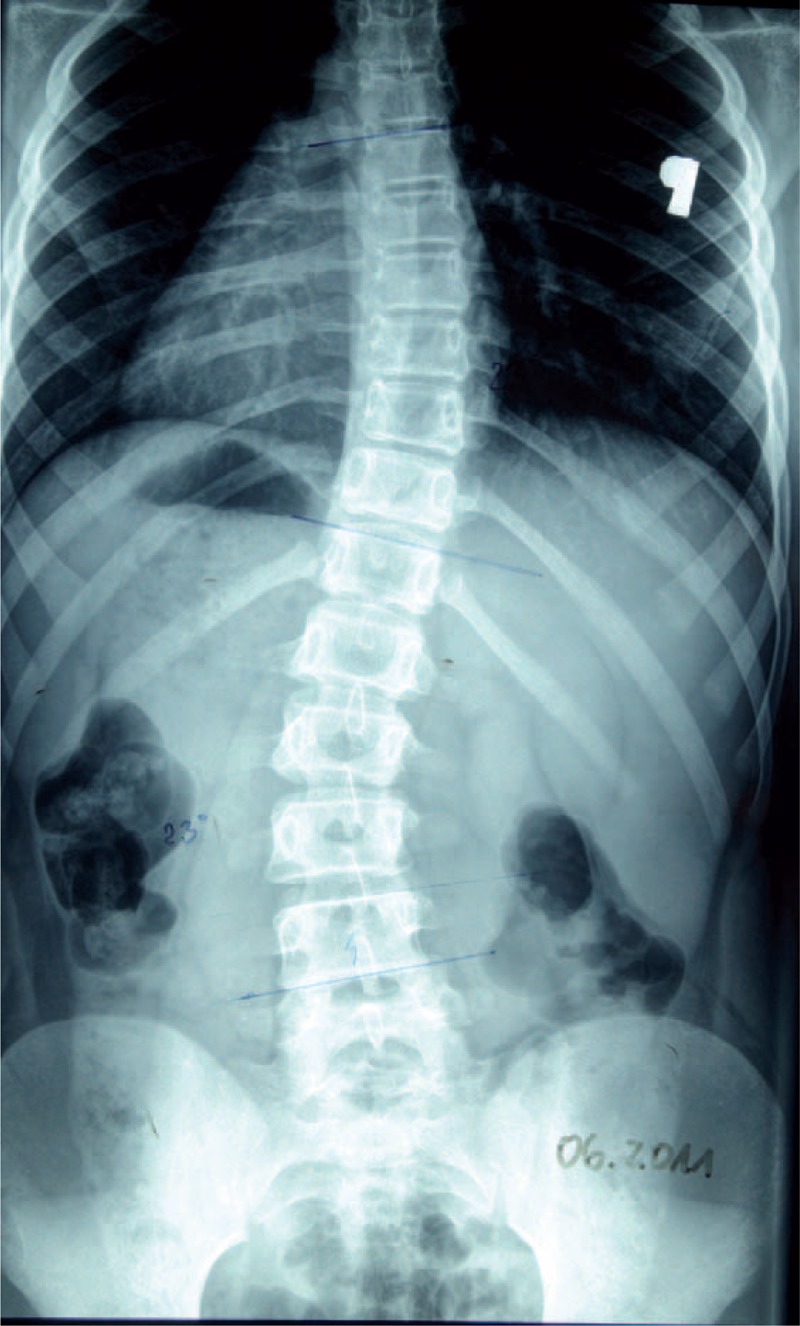
X-ray 06-2011.

**FIGURE 28:**
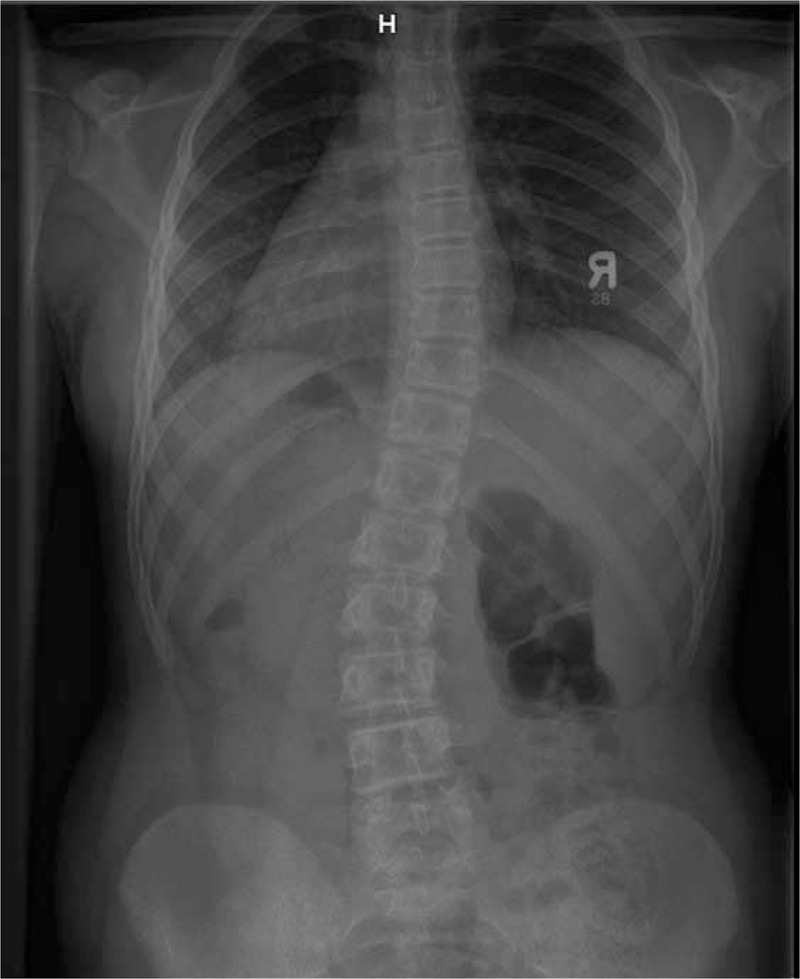
X-ray 06-2012.

**FIGURE 29:**
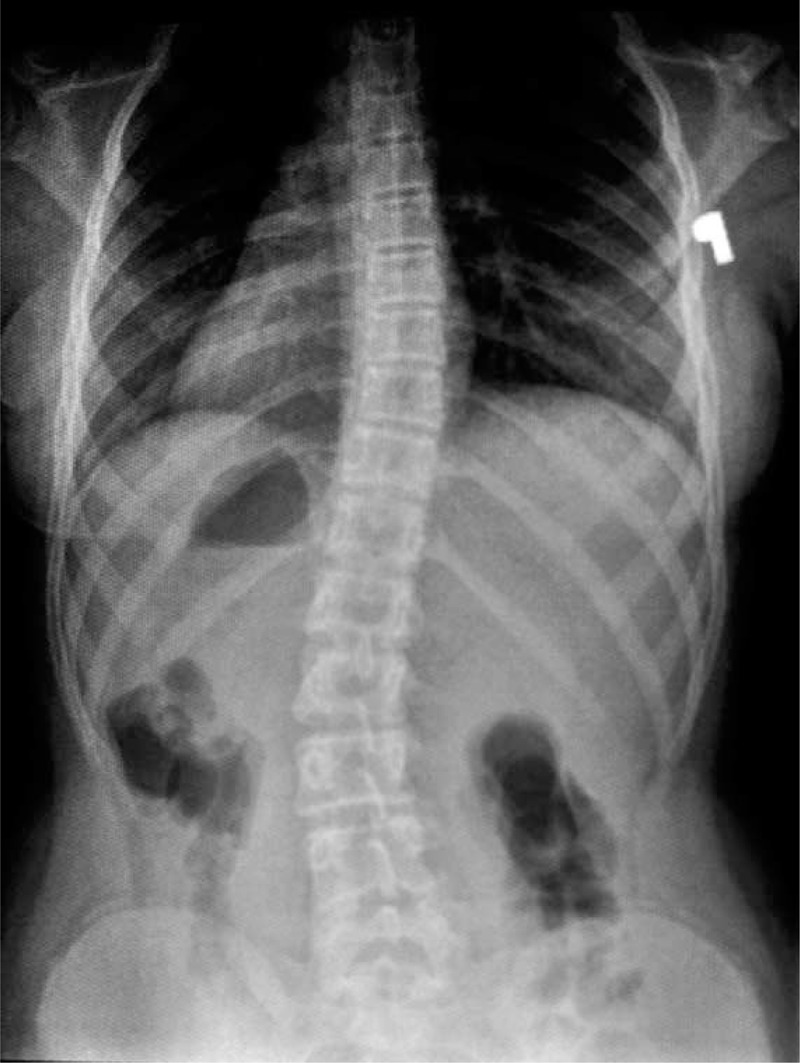
X-ray 05-2013.

**FIGURE 30:**
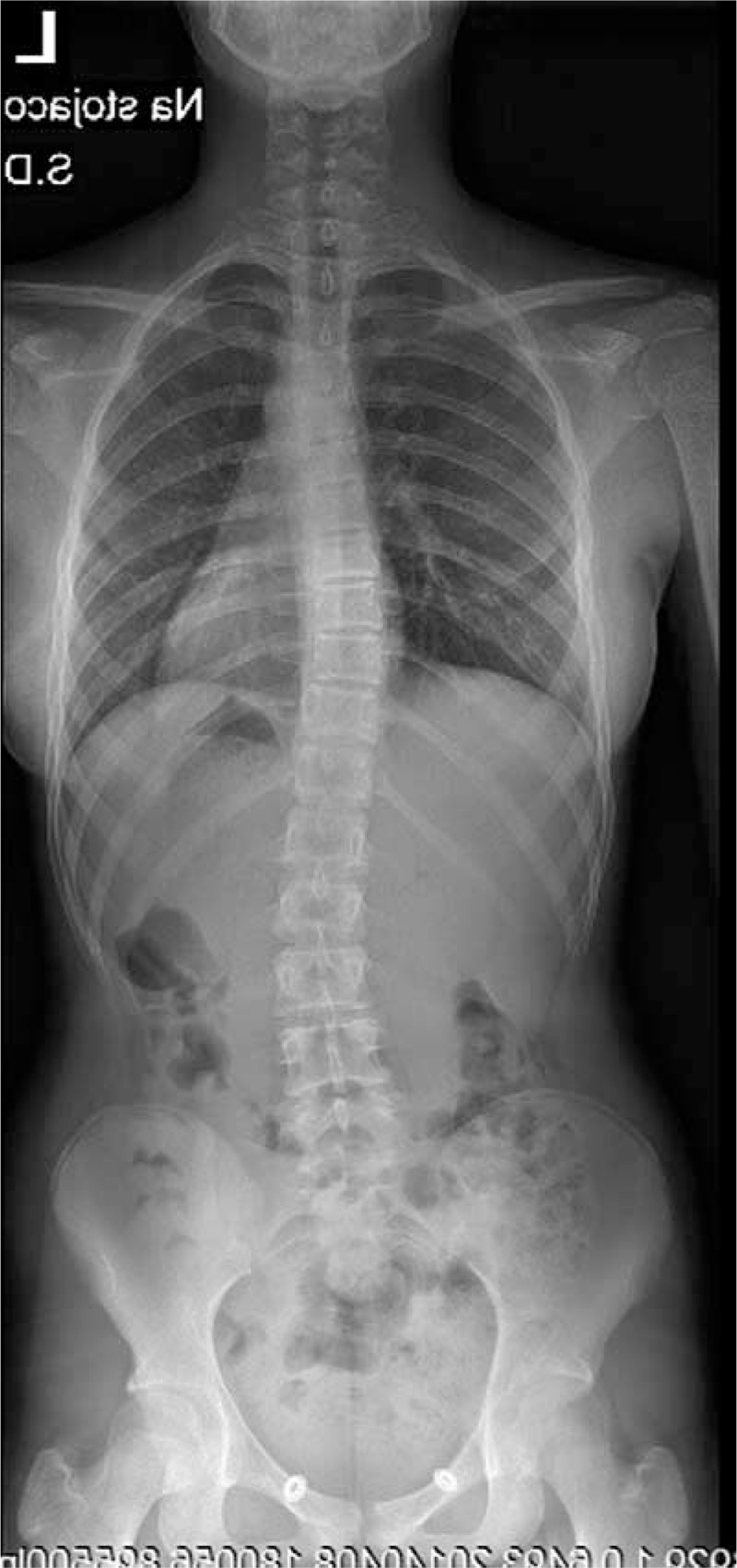
X-ray 04-2014.

**FIGURE 31:**
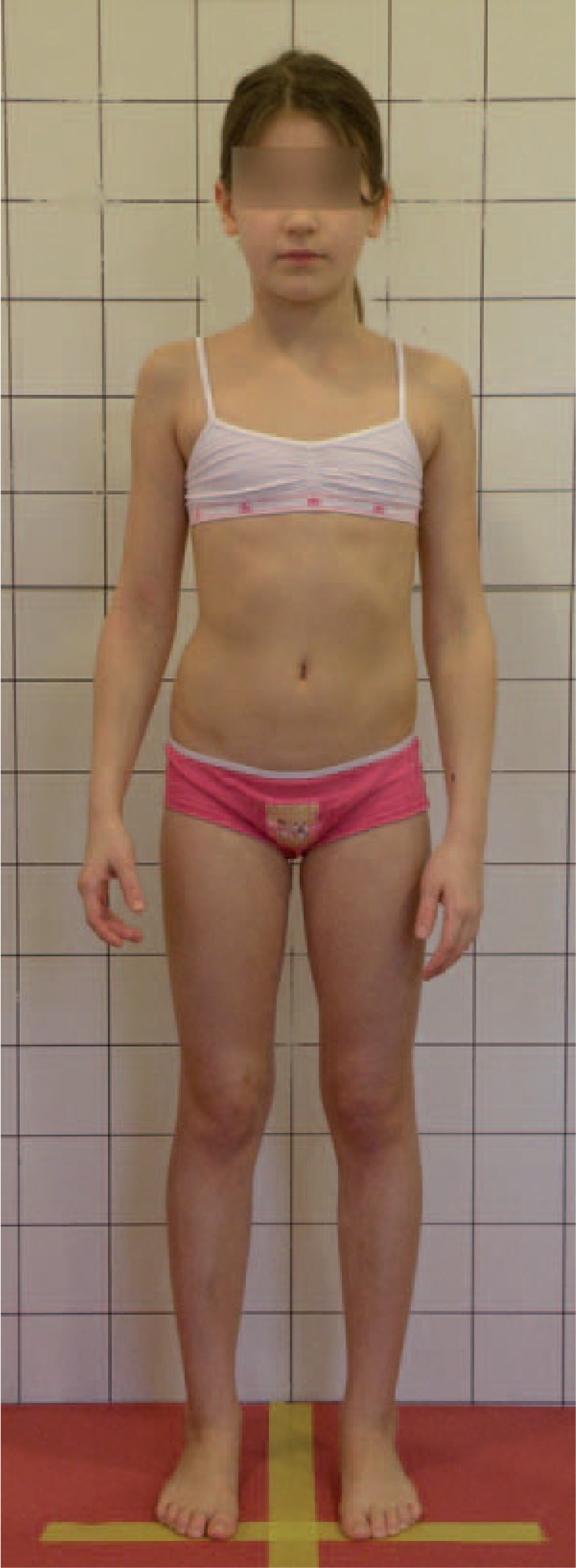
On the front.

**FIGURE 32:**
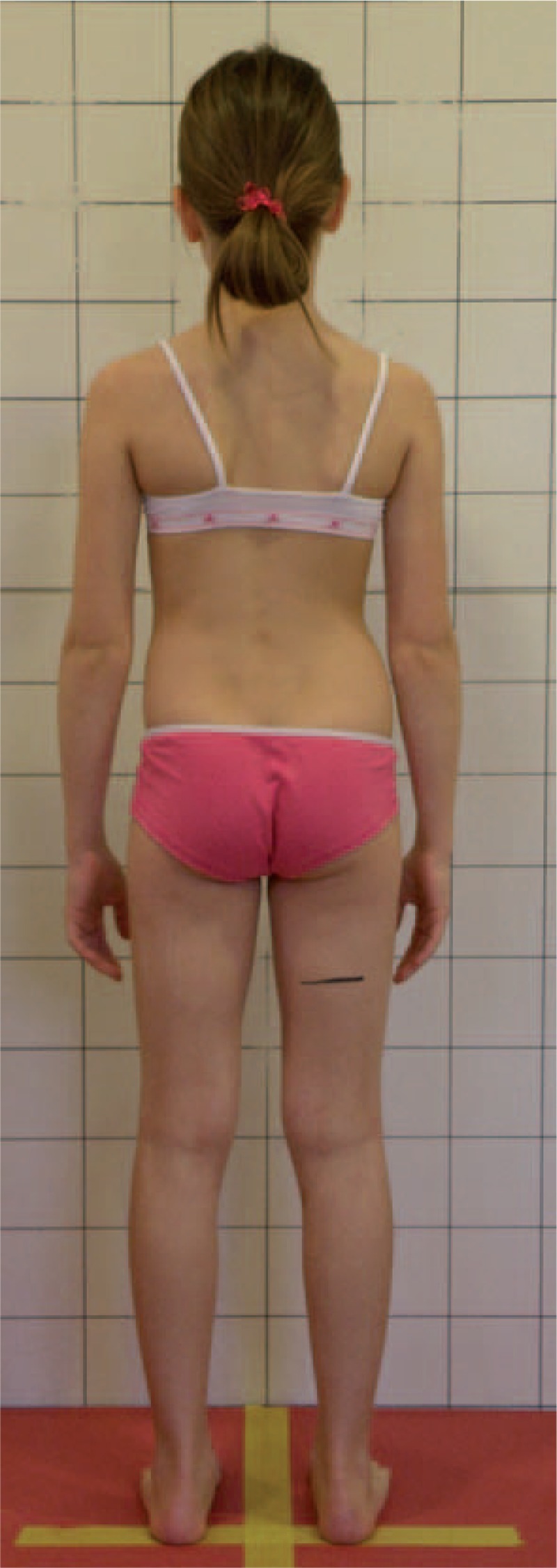
On the back.

**FIGURE 33:**
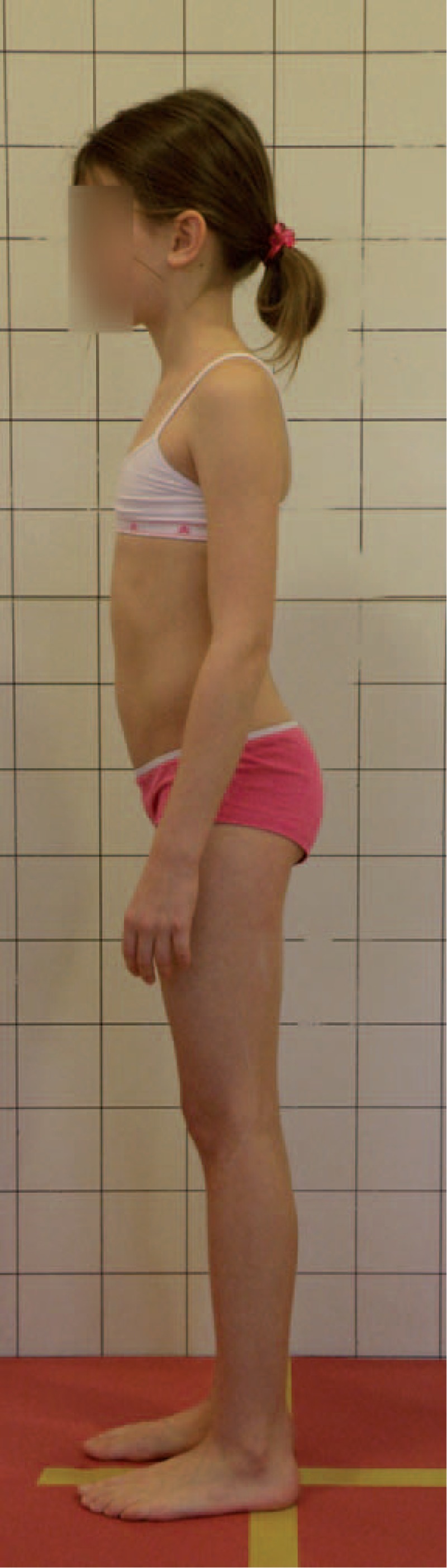
On the side.

**FIGURE 34:**
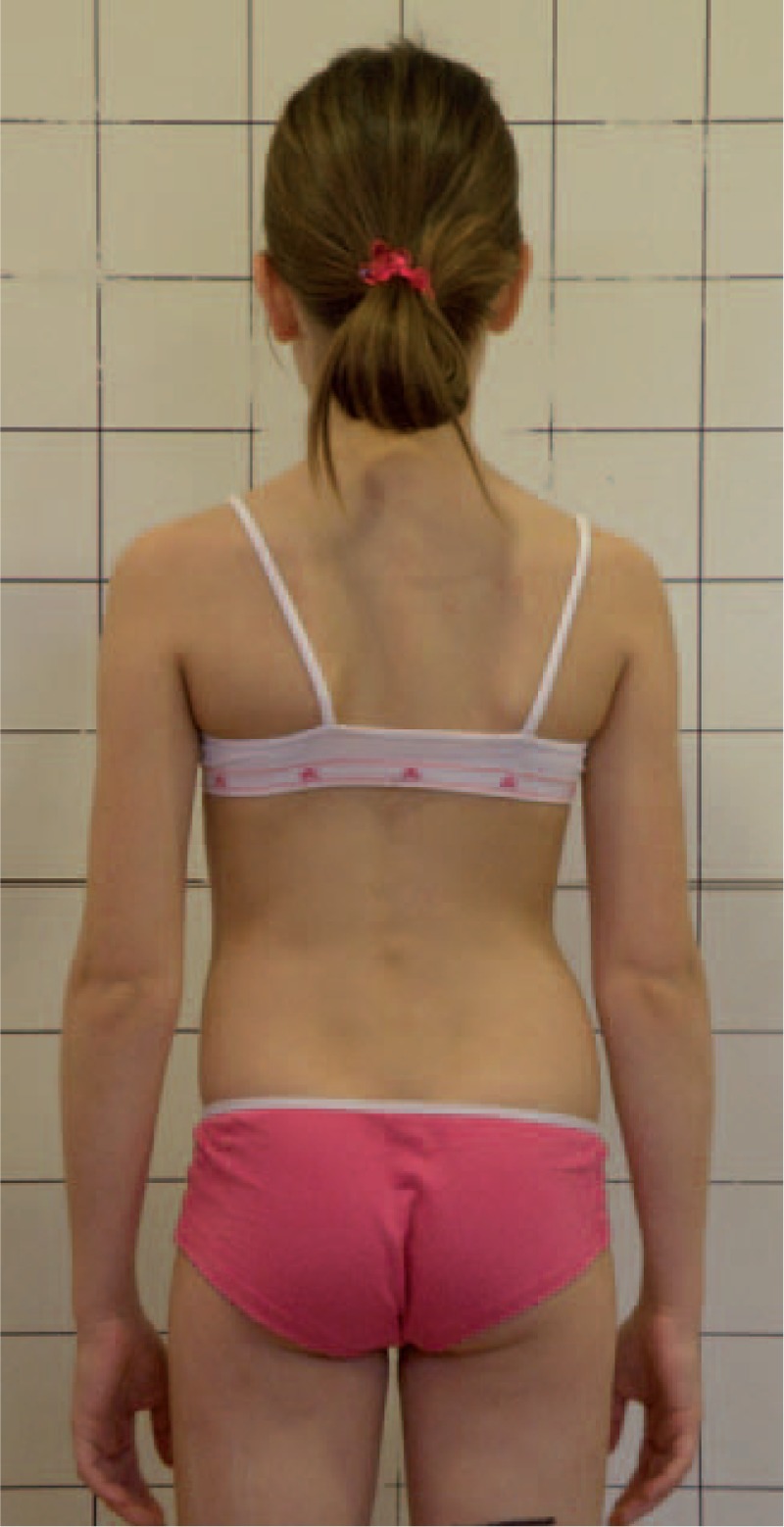
On the back - zoom.

Figures 31–34 show the clinical appearance of 8-year-old girl, Aleksandra before FITS therapy.

Figures 35–42 present clinical appearance of Aleksandra after FITS therapy, at the age of 14 years, 2 years after menarche.

**FIGURE 35:**
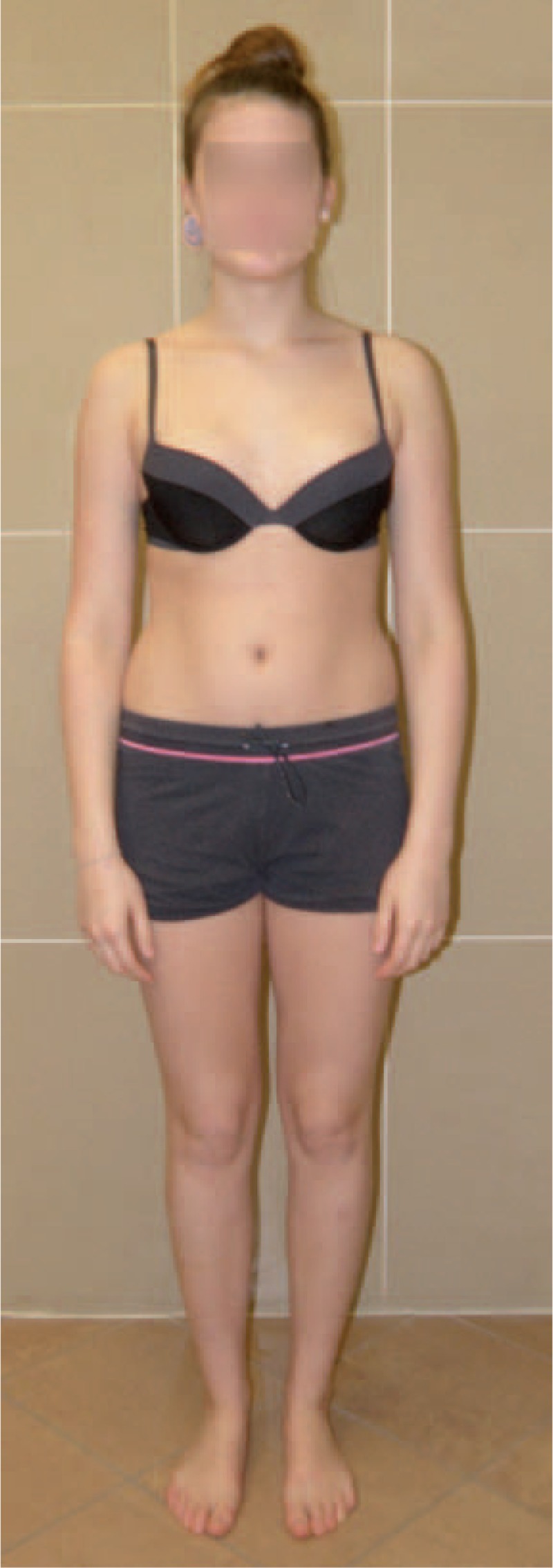
On the front.

**FIGURE 36:**
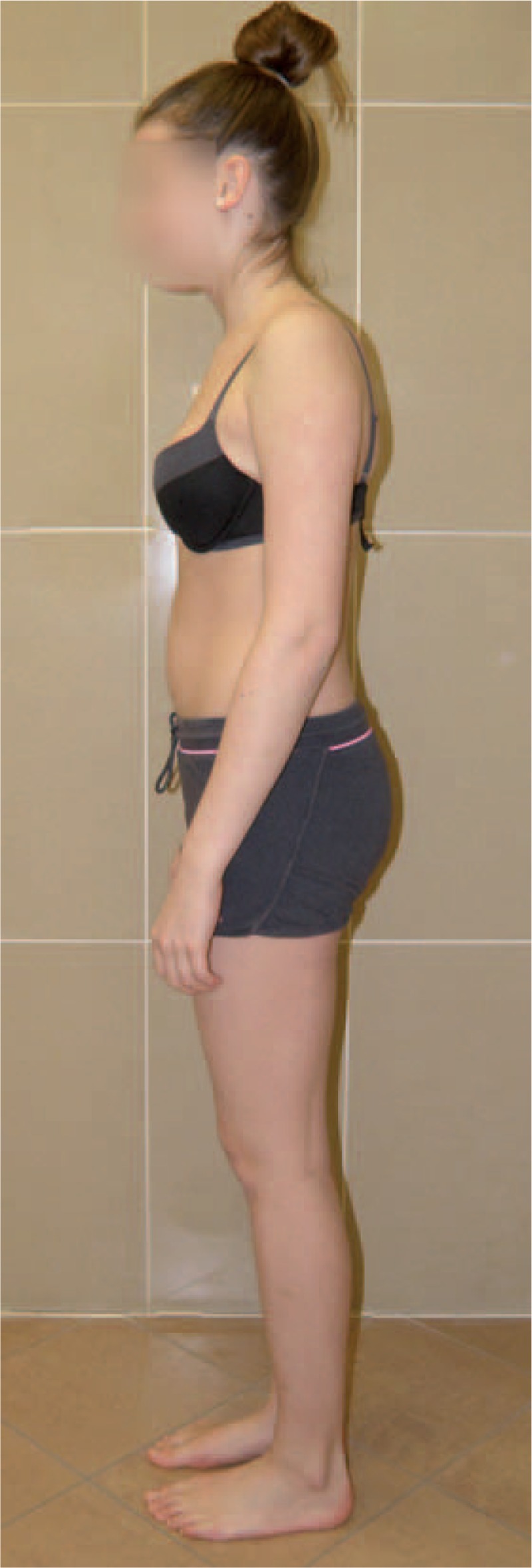
On the side.

**FIGURE 37:**
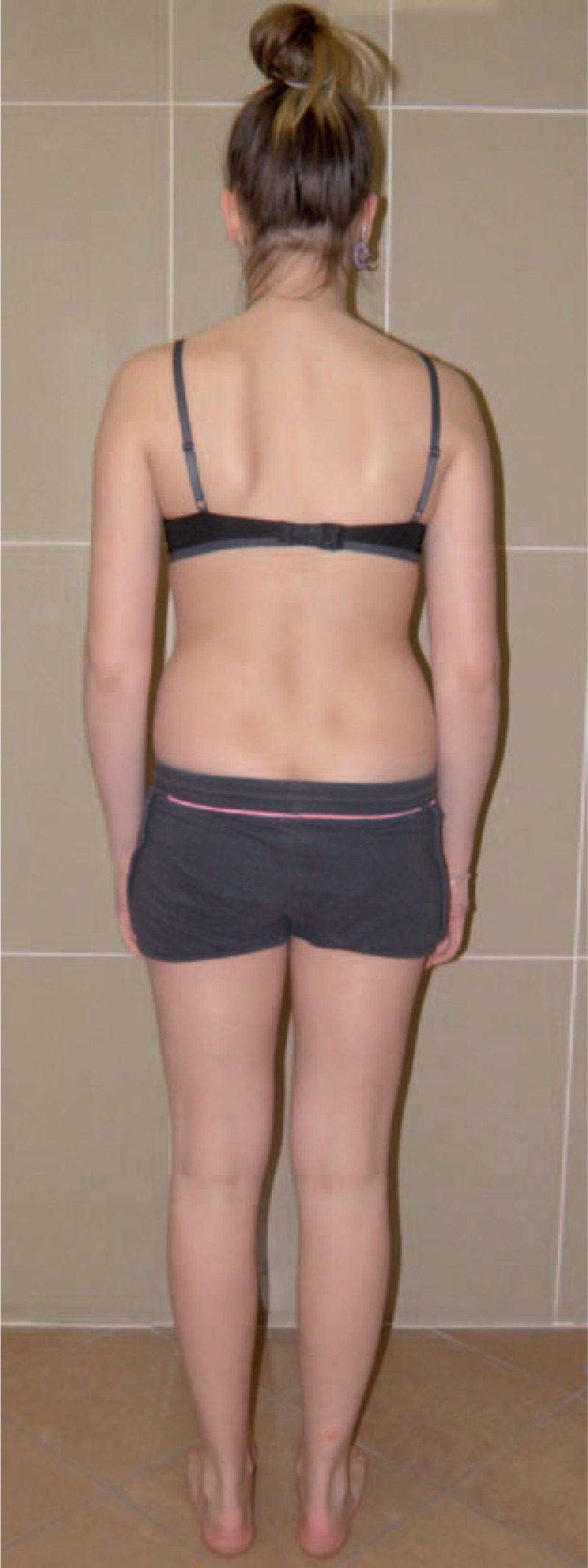
On the back.

**FIGURE 38:**
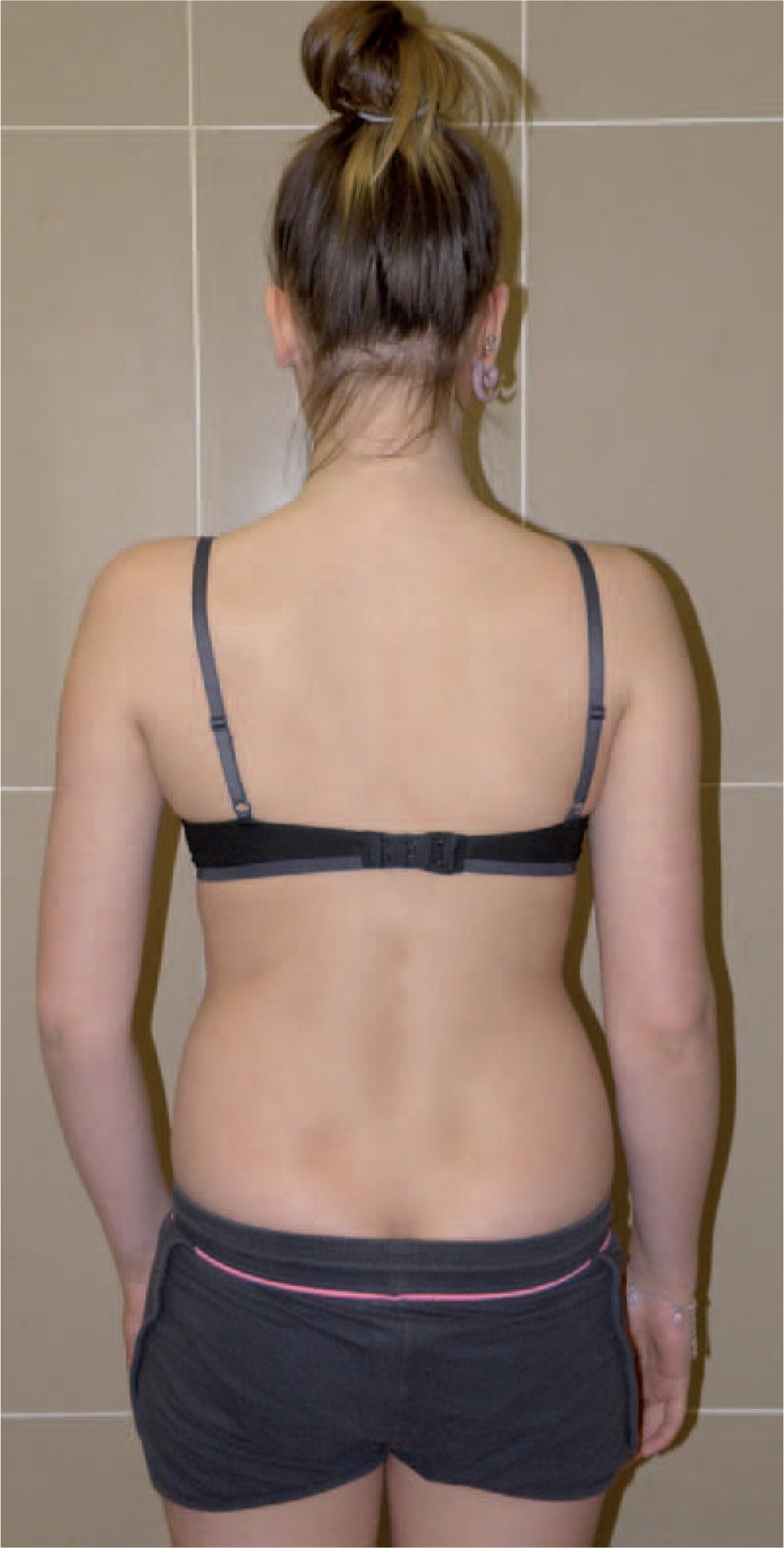
On the back—zoom.

**FIGURE 39:**
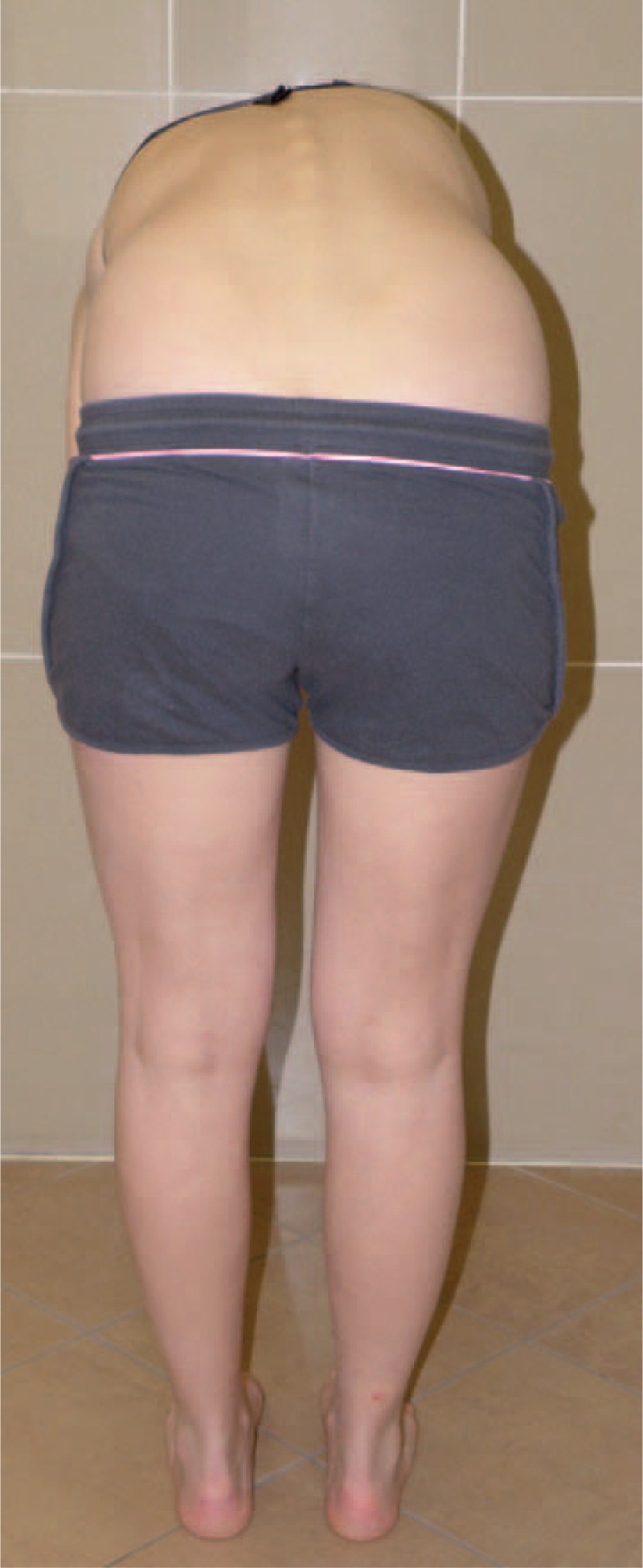
On the back in bending position.

**FIGURE 40:**
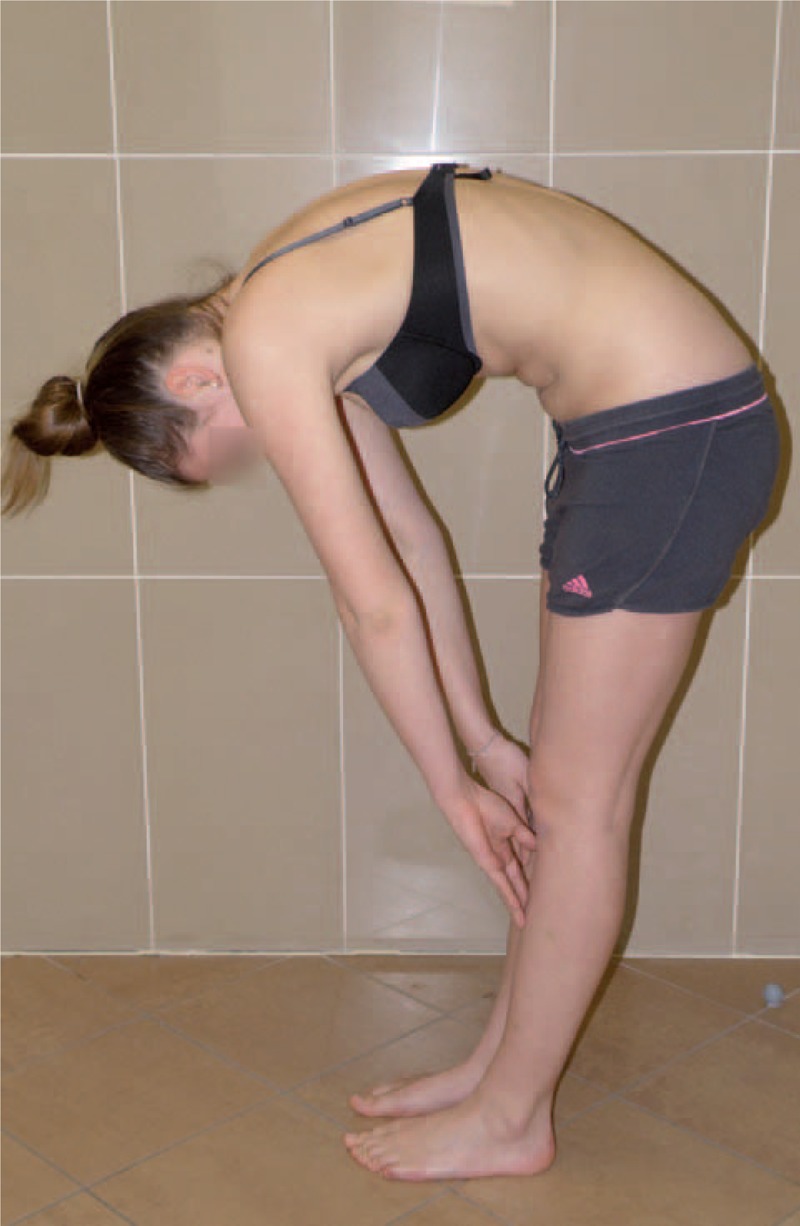
On the side in bending position.

**FIGURE 41:**
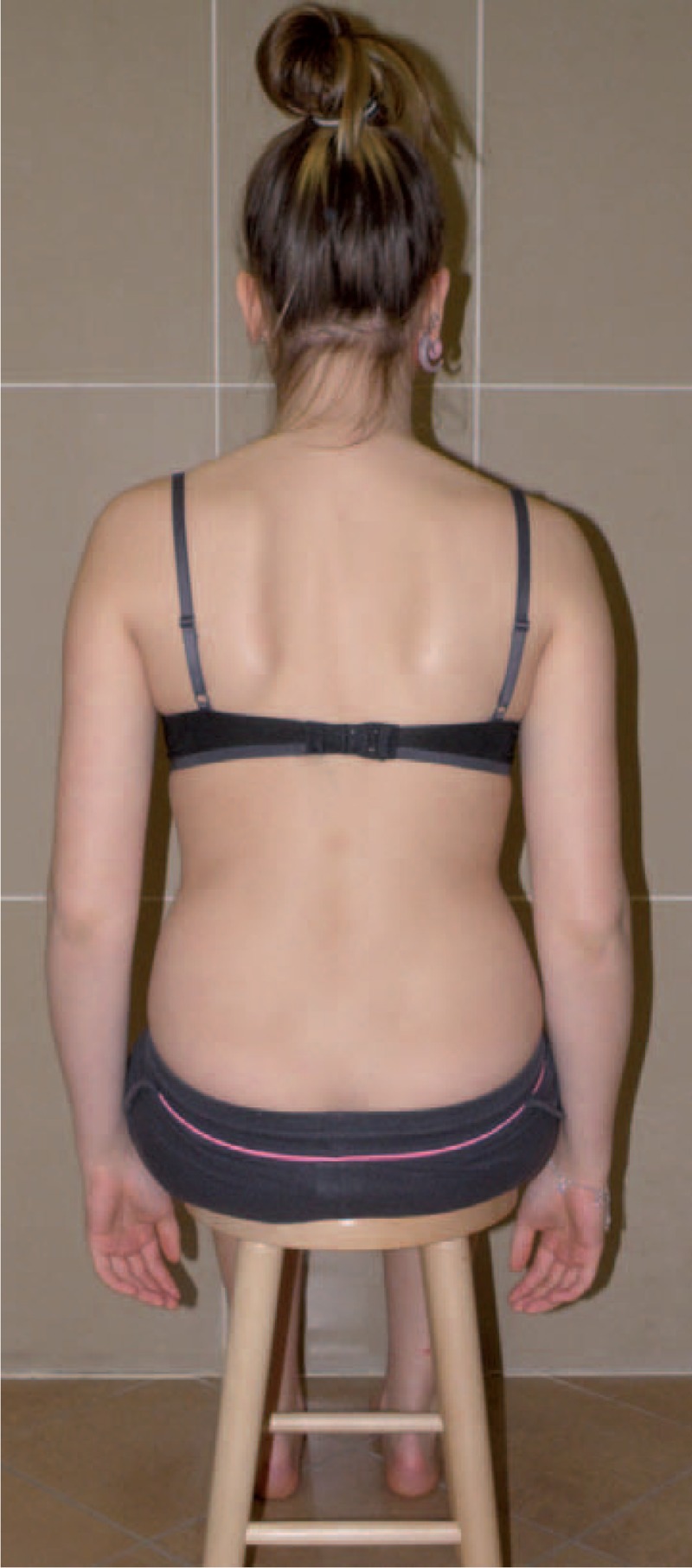
On the back in sitting position without the correction.

**FIGURE 42:**
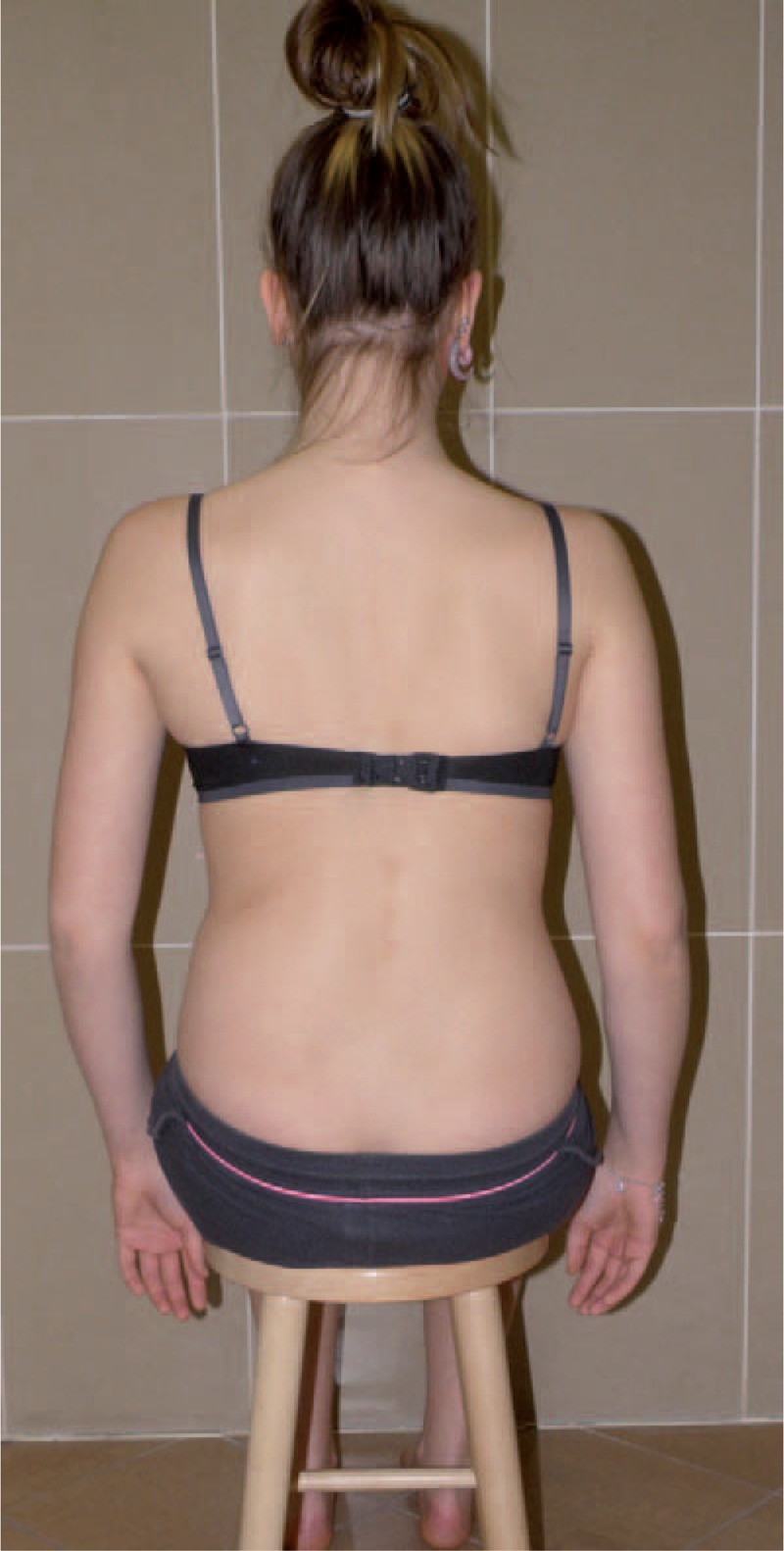
On the back in sitting position in correction.

## DISCUSSION

This study aimed to analyze the children with idiopathic scoliosis of early onset below the age of 10 years who were managed with FITS method. All children were treated under control of one physiotherapist (author). All presented a mild angle structural idiopathic scoliosis with clinical signs of the disease, namely the trunk rotation and the trunk imbalance. Functional scoliosis due to pelvic obliquity, postural muscles weakness, or posture maintenance insufficiency were excluded. Radiographs were ordered and examined by doctors, specialists in orthopedics.

FITS physiotherapy was performed at regular basis of exercises with physiotherapist. The role of parents in assuring regular at home exercises was extremely important. Surprizingly, many young children easily and rapidly learnt exercises, especially stabilizing ones, better than 3D corrective ones. They learnt more easily in supine position having good contact of the back with the ground. Comparing with adolescents the young children got tired earlier and presented less muscular force, on the other hand their concentration revealed sufficient and often better than that in adolescents.

In young children under the age of 10 years the onset and development of idiopathic scoliosis takes place simultaneously with the development of the posture of the child. This is why we can observe the signs of scoliosis together with the signs of postural insufficiency or postural faults. For this reason it seems logical to take into consideration postural aspects when treating children for idiopathic scoliosis. Otherwise, the application of complex 3D active self-correction would be difficult or even impossible to achieve because the child lacks trunk stability and lower limbs correct alignment to be capable of developing corrective movements of the vertebral column.

In this series, the Cobb angle ranged from 11° to 30°. From the natural history of mild angle EOIS we do know that many of these curves (up to 50%) are not progressive in the immediate course. They require regular diagnostic visit to rule out progression. This implies the risk of overtreatment of children with expensive, time consuming, and potentially unnecessary treatment. Unfortunately, we are unable to predict the future of a particular child with mild scoliosis. On the other hand, once progression is done, we are unable to reduce the Cobb angle with nonsurgical techniques, which can be considered argument for early treatment.

It seems that the clinical examination of the young child with EOIS could exceed the regular evaluation of the trunk deformity approved for scoliosis in order to take into consideration additional parameters, which could measure the trunk muscles (abdominal, glutei) strength, the lower trunk stability, the general physical capacity, and the balance capacities. These parameters are important to ensure effective physiotherapy for EOIS and they are often impaired in young children. In such cases, additional program of physiotherapy directed toward postural improvement, muscles strength improvement, and balance improvement can be justified. We use a term of “good/bad feeling of own body” to shortly describe the above-mentioned factors.

Owing to lack of published data on physiotherapy as exclusive treatment for EOIS (bracing excluded) it was difficult to compare the results with other authors.

## CONCLUSION

FITS physiotherapy was effective in preventing curve progression in children with EOIS. At follow-up the Cobb angle was stable or improved (1 case progressed), the trunk imbalance was less and the trunk rotation diminished. Final postpubertal follow-up data are needed.
