# Mild Angle Early Onset Idiopathic Scoliosis Children Avoid Progression Under FITS Method (Functional Individual Therapy of Scoliosis): Erratum

**DOI:** 10.1097/01.md.0000471262.55536.86

**Published:** 2015-08-28

In the article “Mild Angle Early Onset Idiopathic Scoliosis Children Avoid Progression Under FITS Method (Functional Individual Therapy of Scoliosis)”,^1^ which appeared in Volume 94, Issue 20 of *Medicine*, the abstract was not present in the originally published article. The article has since been corrected online.
